# ﻿Four new species of the genus *Diploderma* Hallowell, 1861 (Squamata, Agamidae) from China

**DOI:** 10.3897/zookeys.1148.97706

**Published:** 2023-02-20

**Authors:** Shuo Liu, Mian Hou, Natalia B. Ananjeva, Dingqi Rao

**Affiliations:** 1 Kunming Natural History Museum of Zoology, Kunming Institute of Zoology, Chinese Academy of Sciences, Yunnan 650223, Kunming, China Kunming Institute of Zoology, Chinese Academy of Sciences Kunming China; 2 Kunming Institute of Zoology, Chinese Academy of Sciences, Kunming, Yunnan 650201, Kunming, China Kunming Natural History Museum of Zoology Kunming China; 3 College of Continuing (Online) Education, Sichuan Normal University, Sichuan 610066, Chengdu, China Sichuan Normal University Chengdu China; 4 Zoological Institute, Russian Academy of Sciences, Universitetskaya nab., 1, St. Petersburg 199034, Russia Zoological Institute, Russian Academy of Sciences St. Petersburg Russia

**Keywords:** Hengduan Mountain Region, ND2, Sichuan, systematic, taxonomy, Yunnan

## Abstract

Four new species of *Diploderma* are described from Sichuan and Yunnan provinces, southwestern China, based on an integrative taxonomic approach, combining morphological and genetic data. The first new species from Danba County, Sichuan Province, is morphologically most similar and phylogenetically closely related to *D.flaviceps*, but it can be diagnosed from the latter by having a relatively much shorter tail and by a genetic distance of 4.4% in the ND2 gene; the second new species from Muli County, Sichuan Province, is phylogenetically closely related to *D.daochengense*, *D.yongshengense*, and *D.yulongense*, but it can be diagnosed from the latter three species by having a pale yellow gular spot and by genetic distances of 5.6–6.7% in the ND2 gene; the third new species from Jiulong County, Sichuan Province, is morphologically most similar and phylogenetically closely related to *D.angustelinea*, but it can be diagnosed from the latter by having a relatively much longer tail and by a genetic distance of 2.8% in the ND2 gene; and the last new species from Weixi County, Yunnan Province, is phylogenetically closely related to *D.aorun*, but it can be diagnosed from the latter by having a pale yellow gular spot and by a genetic distance of 2.9% in the ND2 gene. Our work brings the number of species within the genus *Diploderma* to 46.

## ﻿Introduction

Currently the genus *Diploderma* Hallowell, 1861 comprises 42 recognized species (Cai et al. 2022; Liu et al. 2022; Wang et al. 2022b) which are mainly distributed in the hot-dry river valleys of the Hengduan Mountain Region of southwestern China, and more than half of them were described in the past decade (Wang et al. 2021a, 2022a; Cai et al. 2022; Liu et al. 2022; Uetz et al. 2022).

The Hengduan Mountain Region contains many high mountains and rivers, forming complex hot-dry river valleys in this region, which are suitable for *Diploderma* species to inhabit. Therefore, it is not surprising that numerous new species of *Diploderma* were found in this region. However, due to the complex terrain and inconvenient transportation, there are still many areas of this region that have not been fully investigated for biodiversity.

During our field surveys in Yunnan and Sichuan provinces, China, in 2022, some specimens of *Diploderma* were collected from four different sites in the Hengduan Mountain Region. In terms of morphology, these specimens showed distinct differences to any recognized species of the genus. Furthermore, phylogenetic analyses indicated differentiation of these populations also at a molecular level. By comparing our data with morphological and molecular differentiation within existing species of *Diploderma*, we conclude that these populations have exceeded an intraspecific variation level and thus describe four new species of *Diploderma* herein.

## ﻿Materials and methods

### ﻿Sampling

**Figure 1.**
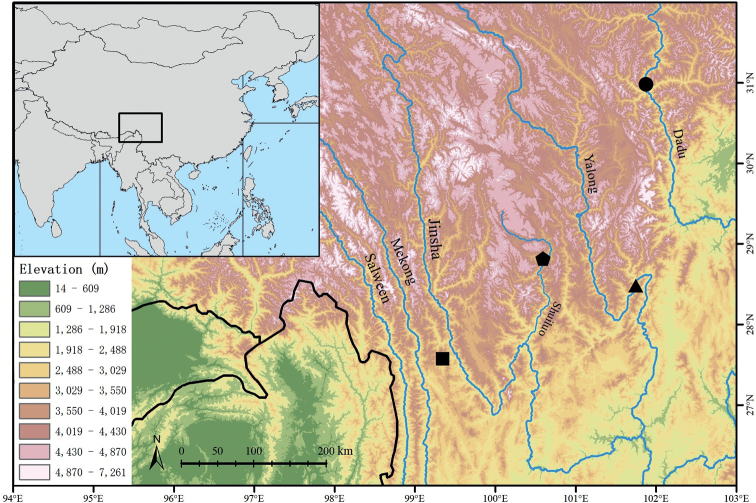
Map showing the type localities of *Diplodermadanbaense* sp. nov. in Danba County, Ganzi Prefecture, Sichuan Province (black dot), *Diplodermadonglangense* sp. nov. in Muli County, Liangshan Prefecture, Sichuan Province (black pentagon), *Diplodermajiulongense* sp. nov. in Jiulong County, Ganzi Prefecture, Sichuan Province (black triangle), and *Diplodermatachengense* sp. nov. in Weixi County, Diqing Prefecture, Yunnan Province (black square), in the Hengduan Mountain Region, southwestern China. The elevation data were obtained from Geospatial Data Cloud (2022).

Field survey in Yunnan Province was conducted from June to July 2022 by Shuo Liu, and field survey in Sichuan Province was conducted from July to August 2022 by Mian Hou. Specimens were collected in the morning or afternoon from Weixi County, Yunnan Province, and Danba, Muli, and Jiulong counties, Sichuan Province, respectively (Fig. 1). Photographs were taken to document colour patterns in life prior to anaesthetisation and euthanasia, respectively. Genetic tissues were stored in 99% ethanol taken from livers, and specimens were preserved in 75% ethanol. All newly collected specimens were deposited at Kunming Natural History Museum of Zoology, Kunming Institute of Zoology, Chinese Academy of Sciences (**KIZ**).

### ﻿Morphological characteristics and analyses

Morphometric measurements and pholidosis data were taken for all newly collected specimens and 27 museum specimens deposited at KIZ. Specimens were measured using a digital caliper to the nearest 0.1 mm. Measurements were taken on the left side (unless the left side was damaged, then the right side was used), values for paired pholidosis characteristics were recorded on both sides and were provided in left/right order (following Liu et al. 2020, 2022; Wang et al. 2022a, b). The following morphometric characteristics were measured and counted following Liu et al. (2022) and Wang et al. (2022a):

**F4S** subdigital lamellae under fourth finger, subdigital lamellae scales from the base between third and fourth finger to the tip of fourth finger, excluding the claw;

**FLL** fore-limb length, measured between the point of insertion at axillary to the tip of fourth finger, excluding the claw, measured on straightened limb;

**HD** head depth, measured as the perpendicular distance at the temporal region of head;

**HL** head length, measured from the tip of snout to the rear border of the angle of jaw;

**HLL** hind limb length, measured between the point of insertion at groin to the tip of fourth toe, excluding the claw, measured on straightened limb;

**HW** head width, measured between the widest points of the head;

**IL** infralabial scale number, enlarged, modified labial scales from mental to the corner of mouth;

**MD** middorsal crest scale number, modified crest scales longitudinally from the first nuchal crest to the scale above cloaca;

**NSL** nasal–supralabials scale rows, number of horizontal rows of small scales between the first supralabial and the nasal;

**SEL** snout–eye length, measured between the tip of snout and anterior edge of orbital bone;

**SL** supralabial scale number, enlarged, modified labial scales from rostral to the corner of mouth;

**SOR** suborbital scale rows, longitudinal rows of scales between supralabials and inferior-most edge of orbit circle, excluding fine ciliary scales in the orbit;

**SVL** snout–vent length, measured from the snout tip to anterior edge of the cloaca;

**T4L** fourth toe length, measured between the tip of fourth toe to the base between third and fourth toe, excluding the claw;

**T4S** subdigital lamellae under fourth toe, subdigital lamellae scales from the base between third and fourth toe to the tip of fourth toe, excluding the claw;

**TAL** tail length, measured from the anterior edge of the cloaca to the tip of tail;

**TRL** trunk length, measured between the limb insertion points between axillary and groin;

**VN** ventral scale number, ventral body scales counted in a straight line along the medial axis between the transverse gular fold and the anterior edge of cloaca.

We compared morphological characters of the new species with other members of the genus relying on examined specimens and original species descriptions (Hallowell 1861; Günther 1864; Anderson 1878; Boulenger 1906, 1918; Barbour and Dunn 1919; Stejneger 1924; Mertens 1926; Smith 1935; Gressitt 1936; Bourret 1937; Song 1987; Ota 1989; Ota et al. 1998; Li et al. 2001; Gao and Hou 2002; Manthey et al. 2012; Wang et al. 2015, 2016, 2019b, d, 2021a, b, 2022a; Ananjeva et al. 2017; Rao et al. 2017; Liu et al. 2020, 2022; Cai et al. 2022), and the additional data from Wu et al. (2005), Manthey (2008), and Wang et al. (2017, 2018, 2019b, c, 2021a, 2022b).

Principal component analysis (PCA) was used to determine whether the newly collected specimens and species with closely morphological similarities to them occupied unique positions in morphospace, and whether the results coincide with the delineation of species as indicated by molecular phylogenetic analysis. Characters used in the PCA were mensural data from SVL, TAL, HL, HW, HD, SEL, FLL, HLL, TRL, and T4L. PCA was performed using the prcomp command in R 4.2.2 (R Core Team 2022). The scatter plot was plotted using the ggplot2 package in R 4.2.2. As species of *Diploderma* are known to be sexually dimorphic in morphometric measurements (Manthey et al. 2012; Wang et al. 2015, 2018, 2019b), mensural data of each sex are treated separately for analyses.

### ﻿Molecular analysis

Total genomic DNA for the newly collected specimens was extracted from liver tissues with a standard three step phenol chloroform extraction method (Sambrook et al. 1989). The mitochondrial gene NADH dehydrogenase subunit 2 (ND2) was amplified and sequenced by using published primers (Wang et al. 2019a). PCR and sequencing methods were the same as Liu et al. (2020). All new sequences were edited and manually managed using SeqMan in Lasergene 7.1 (DNASTAR Inc., Madison, WI, USA) and MEGA 11 (Tamura et al. 2021). Representative species of *Pseudocalotes* Fitzinger were chosen as outgroups (Liu et al. 2022). Genetic data for 39 species of *Diploderma* and two species of outgroup taxa were obtained from GenBank (Table 1). The technical computation methods for sequences alignment, best substitution model selection, Bayesian inference (BI) and maximum likelihood (ML) phylogenetic analyses, and genetic divergences calculation were the same as those in Liu et al. (2022).

**Table 1.** GenBank accession numbers for the sequences used in this study.

Species	Voucher	Locality	Accession
*Diplodermaangustelinea*	KIZ 029704	Muli, Sichuan, China	MT577930
	KIZ 029705	Muli, Sichuan, China	MT577924
	KIZ 029708	Muli, Sichuan, China	MT577931
	KIZ 029710	Muli, Sichuan, China	MT577927
	KIZ 032488	Muli, Sichuan, China	MT577925
	KIZ 032489	Muli, Sichuan, China	MT577926
	KIZ 032490	Muli, Sichuan, China	MT577928
	KIZ 032491	Muli, Sichuan, China	MT577929
*Diplodermaaorun*	KIZ 032733	Benzilan, Yunnan, China	MT577938
	KIZ 032734	Benzilan, Yunnan, China	MT577939
	KIZ 032735	Benzilan, Yunnan, China	MT577937
	KIZ 032736	Benzilan, Yunnan, China	MT577936
	KIZ 032737	Benzilan, Yunnan, China	MT577940
*Diplodermabatangense*	KIZ 09404	Zhubalong, Tibet, China	MK001412
	KIZ 019276	Batang, Sichuan, China	MK001413
*Diplodermabrevicauda*	KIZ 044305	Lijiang, Yunnan, China	MW506021
	KIZ 044306	Lijiang, Yunnan, China	MW506022
*Diplodermabowoense*	KIZ 044757	Muli, Sichuan, China	MW506020
	KIZ 044758	Muli, Sichuan, China	MW506019
*Diplodermabrevipes*	NMNS 19607	Taiwan, China	MK001429
	NMNS 19608	Taiwan, China	MK001430
*Diplodermachapaense*	KIZ 034923	Lvchun, Yunnan, China	MG214263
	ZMMU NAP-01911	Chapa, Vietnam	MG214262
*Diplodermadaochengense*	20210905	Muli, Sichuan, China	OP595620
	DC001	Daocheng, Sichuan, China	OP595621
	DC003	Daocheng, Sichuan, China	OP595622
	DC004	Daocheng, Sichuan, China	OP595623
	20210904	Muli, Sichuan, China	OP595624
*Diplodermadrukdaypo*	KIZ 027627	Jinduo, Tibet, China	MT577950
	KIZ 027628	Zhuka, Tibet, China	MT577952
*Diplodermadymondi*	KIZ 040639	Dongchuan, Yunnan, China	MK001422
	KIZ 040640	Dongchuan, Yunnan, China	MK001423
*Diplodermafasciatum*	SYS r002175	Wuming, Guangxi, China	OM055809
	KIZ 040192	Daweishan, Yunnan, China	OM055800
*Diplodermaflaviceps*	KIZ 01851	Luding, Sichuan, China	MK001416
	KIZ 01852	Luding, Sichuan, China	MK001417
	KIZ 019575	Kangding, Sichuan, China	MT577896
	KIZ 019576	Kangding, Sichuan, China	MT577897
	KIZ 019577	Kangding, Sichuan, China	MT577895
	KIZ 019578	Kangding, Sichuan, China	MT577894
	KIZ 019579	Kangding, Sichuan, China	MT577898
*Diplodermaflavilabre*	KIZ 032692	Baiyu,Sichuan, China	MT577916
	KIZ 032694	Baiyu,Sichuan, China	MT577917
*Diplodermaformosgulae*	KIZ 044420	Deqin, Yunnan, China	MW506024
	KIZ 044421	Deqin, Yunnan, China	MW506025
*Diplodermaiadinum*	KIZ 027697	Yunling, Yunnan, China	MT577956
	KIZ 027702	Yunling, Yunnan, China	MT577957
*Diplodermakangdingense*	20210916	Kangding, Sichuan, China	OP595625
	20210917	Kangding, Sichuan, China	OP595626
*Diplodermalaeviventre*	KIZ 014037	Basu, Tibet, China	MK001407
	KIZ 027691	Basu, Tibet, China	MT577892
*Diplodermalimingense*	KIZ2022015	Yulong, Yunnan, China	OP428783
	KIZ2022017	Yulong, Yunnan, China	OP428784
*Diplodermaluei*	NMNS 19604	Taiwan, China	MK001433
	NMNS 19605	Taiwan, China	MK001434
*Diplodermamakii*	NMNS 19609	Taiwan, China	MK001431
	NMNH 19610	Taiwan, China	MK001432
*Diplodermamenghaiense*	KIZ L0030	Menghai, Yunnan, China	MT598655
	KIZ L0031	Menghai, Yunnan, China	MT598656
*Diplodermamicangshanense*	KIZ 032801	Shiyan, Hubei, China	MK578665
	KIZ 023231	Xixia, Henan, China	MK578664
*Diplodermapanchi*	KIZ 032715	Yajiang, Sichuan, China	MT577946
	KIZ 032716	Yajiang, Sichuan, China	MT577944
*Diplodermapanlong*	KIZ 040137	Miansha, Sichuan, China	MT577906
	KIZ 040138	Miansha, Sichuan, China	MT577907
*Diplodermapolygonatum*	NMNS 19598	Taiwan, China	MK001427
	NMNS 19599	Taiwan, China	MK001428
*Diplodermaqilin*	KIZ 028332	Deqin, Yunnan, China	MT577941
	KIZ 028333	Deqin, Yunnan, China	MT577942
*Diplodermashuoquense*	KIZ2022004	Xiangcheng, Sichuan, China	OP428773
	KIZ2022005	Xiangcheng, Sichuan, China	OP428774
*Diplodermaslowinskii*	CAS 214906	Gongshan, Yunnan, China	MK001405
	CAS 214954	Gongshan, Yunnan, China	MK001406
*Diplodermasplendidum*	KIZ 015973	Yichang, Hubei, China	MK001418
	LSUMZ 81212	Unknown	AF288230
*Diplodermaswild*	KIZ 034914	Panzhihua, Sichuan, China	MN266299
	KIZ 034894	Panzhihua, Sichuan, China	MN266300
*Diplodermaswinhonis*	NMNS 19592	Taiwan, China	MK001419
	NMNS 19593	Taiwan, China	MK001420
*Diplodermavarcoe*	WK-JK 011	Yuxi, Yunnan, China	MT577903
	KIZ 026132	Mengzi, Yunnan, China	MK001421
*Diplodermavela*	KIZ 019299	Quzika, Tibet, China	MK001414
	KIZ 034925	Quzika, Tibet, China	MK001415
*Diplodermaxinlongense*	20210907	Xinlong, Sichuan, China	OP595613
	20210908	Xinlong, Sichuan, China	OP595614
*Diplodermayangi*	SWFU 005410	Chayu, Tibet, China	OL449603
	SWFU 005412	Chayu, Tibet, China	OL449604
*Diplodermayongshengense*	KIZ2022009	Yongsheng, Yunnan, China	OP428777
	KIZ2022008	Yongsheng, Yunnan, China	OP428778
	KIZ2022010	Yongsheng, Yunnan, China	OP428779
	KIZ2022011	Yongsheng, Yunnan, China	OP428780
*Diplodermayulongense*	KIZ 028291	Hutiaoxia, Yunnan, China	MT577921
	KIZ 028292	Hutiaoxia, Yunnan, China	MT577922
	KIZ 028300	Baishuitai, Yunnan, China	MT577923
	KIZ 09399	Xianggelila, Yunnan, China	MK001410
	KIZ 043196	Xianggelila, Yunnan, China	MK001411
*Diplodermayunnanense*	CAS 242271	Baoshan, Yunnan, China	MK001408
	KIZ 040193	Yingjiang, Yunnan, China	MK578658
*Diplodermazhaoermii*	KIZ 019564	Wenchuan, Sichuan, China	MK001425
	KIZ 019565	Wenchuan, Sichuan, China	MK001426
*Diplodermadanbaense* sp. nov.	KIZ2022048	Danba, Sichuan, China	OQ378180
	KIZ2022049	Danba, Sichuan, China	OQ378181
	KIZ2022050	Danba, Sichuan, China	OQ378182
	KIZ2022051	Danba, Sichuan, China	OQ378183
	KIZ2022056	Danba, Sichuan, China	OQ378184
*Diplodermadonglangense* sp. nov.	KIZ2022057	Muli, Sichuan, China	OQ378185
	KIZ2022058	Muli, Sichuan, China	OQ378186
	KIZ2022059	Muli, Sichuan, China	OQ378187
	KIZ2022060	Muli, Sichuan, China	OQ378188
	KIZ2022061	Muli, Sichuan, China	OQ378189
*Diplodermajiulongense* sp. nov.	KIZ2022086	Jiulong, Sichuan, China	OQ378190
	KIZ2022087	Jiulong, Sichuan, China	OQ378191
	KIZ2022099	Jiulong, Sichuan, China	OQ378192
	KIZ2022100	Jiulong, Sichuan, China	OQ378193
	KIZ2022101	Jiulong, Sichuan, China	OQ378194
*Diplodermatachengense* sp. nov.	KIZ2022028	Weixi, Yunnan, China	OQ378195
	KIZ2022027	Weixi, Yunnan, China	OQ378196
	KIZ2022029	Weixi, Yunnan, China	OQ378197
	KIZ2022038	Weixi, Yunnan, China	OQ378198
	KIZ2022039	Weixi, Yunnan, China	OQ378199
*Pseudocalotesbrevipes*	MVZ 224106	Vinh Phuc, Vietnam	AF128502
*Pseudocaloteskakhiensis*	KIZ 015975	Gongshan, Yunnan, China	MK001435

## ﻿Results

**Figure 2.**
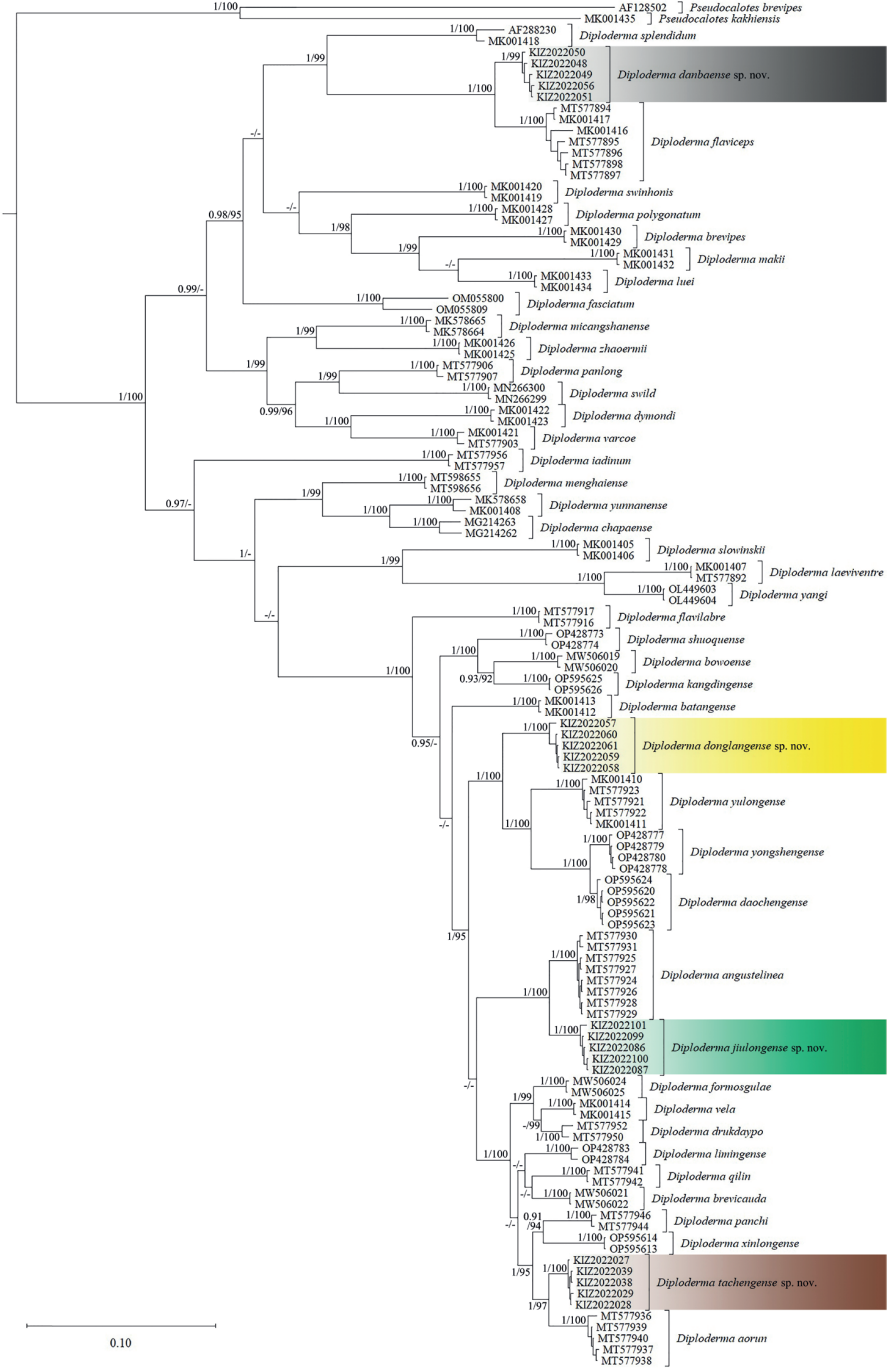
Bayesian phylogram of the genus *Diploderma* inferred from mitochondrial gene ND2 (1028 bp). Numbers before slashes indicate Bayesian posterior probabilities and numbers after slashes indicate ML ultrafast bootstrap values. The symbol “–” represents the value below 0.90/90.

The obtained sequence alignment is 1028 bp in length. The resulting topologies from BI and ML analyses are consistent (Fig. 2). The specimens from Danba County formed a clade sister to *Diplodermaflaviceps* (Barbour & Dunn, 1919) with strong support; the specimens from Muli County formed a clade sister to the clade consisting of *D.daochengense* Cai, Zhang, Li, Du, Xie, Hou, Zhou & Jiang, 2022, *D.yongshengense* Liu, Hou, Rao & Ananjeva, 2022, and *D.yulongense* (Manthey, Denzer, Hou & Wang, 2012) with strong support; the specimens from Jiulong County formed a clade sister to *D.angustelinea* Wang, Ren, Wu, Che & Siler, 2020 with strong support; and the specimens from Weixi County formed a clade sister to *D.aorun* Wang, Jiang, Zheng, Xie, Che & Siler, 2020 with strong support. The minimum uncorrected pairwise distance between the specimens from Danba County and other species of *Diploderma* is 4.4% (between the population from Danba County and *D.flaviceps*), the minimum uncorrected pairwise distance between the specimens from Muli County and other species of *Diploderma* is 5.6% (between the population from Muli County and *D.yulongense*), the minimum uncorrected pairwise distance between the specimens from Jiulong County and other species of *Diploderma* is 2.8% (between the population from Jiulong County and *D.angustelinea*), and the minimum uncorrected pairwise distance between the specimens from Weixi County and other species of *Diploderma* is 2.9% (between the population from Weixi County and *D.aorun*), which are all greater than the genetic distance (2.6%) between the two recognized species *D.drukdaypo* and *D.vela* (Suppl. material 1).

**Figure 3.**
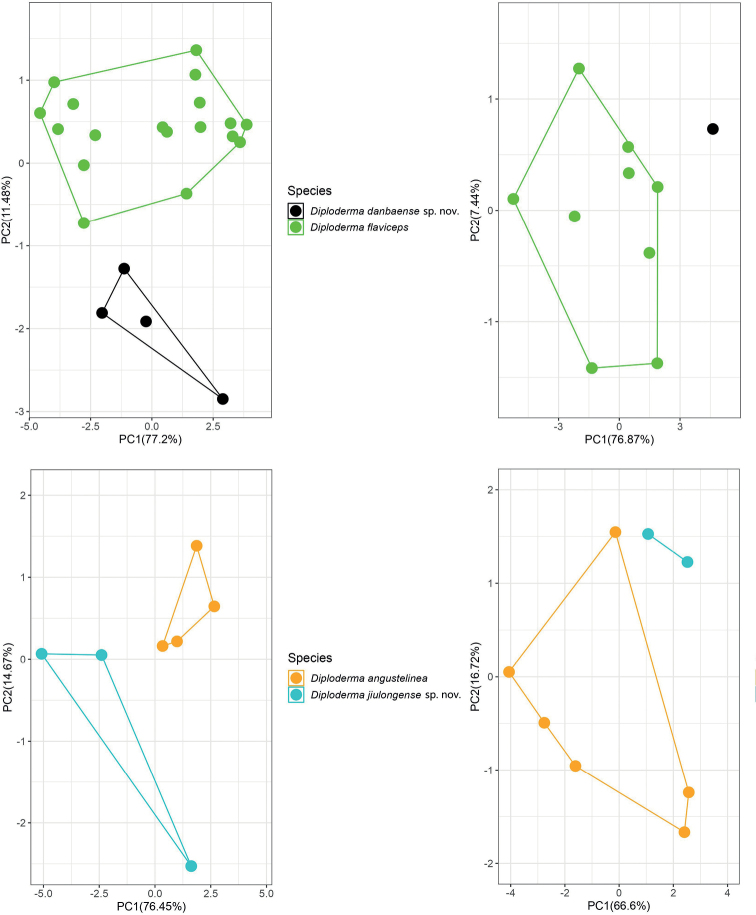
PCA based on ten morphometric characteristics (SVL, TAL, HL, HW, HD, SEL, FLL, HLL, TRL, and T4L) for males of *Diplodermaflaviceps* and *Diplodermadanbaense* sp. nov. (upper left), females of *D.flaviceps* and *Diplodermadanbaense* sp. nov. (upper right), males of *D.angustelinea* and *Diplodermajiulongense* sp. nov. (lower left), and females of *D.angustelinea* and *Diplodermajiulongense* sp. nov. (lower right).

The first two major principal components from PCA were retained, which accounted for 83.32%–91.12% of the total variances. The scatter plot based on PC1 and PC2 showed that the newly collected specimens and species with closely morphological similarities to them can be segregated in both sexes (Fig. 3).

## ﻿Taxonomy

### ﻿ Diploderma danbaense sp. nov.

Taxon classificationAnimaliaSquamataAgamidae

D4D5BA17-1CE2-5B0D-8CD2-3E1B62511B32

https://zoobank.org/08AA2744-059D-4295-AAEC-617A595FC3C0

Figs 4 , 5 , 6

#### Type material.

***Holotype*.** KIZ2022048, adult male, collected on 6 August 2022 by Mian Hou from Bawang Township, Danba County, Ganzi Prefecture, Sichuan Province, China (30°58'59"N, 101°52'29"E, 2020 m elevation).

***Paratypes*.** KIZ2022049, KIZ2022050, KIZ2022056, three adult males; KIZ2022051, one adult female; collecting information the same as the holotype.

#### Etymology.

The specific epithet refers to Danba County, where the new species was discovered.

#### Diagnosis.

*Diplodermadanbaense* sp. nov. can be diagnosed from congeners by a combination of the following morphological characteristics: (1) body size large, SVL 68.7–77.0 mm in adult males, 76.6 in adult female; (2) tail short, TAL/SVL 1.61–1.78 in adult males, 1.55 in adult female; (3) head relatively long, HW/HL 0.66–0.75 in adult males, 0.63 in adult female; (4) limbs moderately long, FLL/SVL 0.41–0.47 in adult males, 0.44 in adult female, HLL/SVL 0.66–0.70 in adult males, 0.65 in adult female; (5) MD 48–58; (6) F4S 16–20, T4S 21–26; (7) tympanum concealed; (8) nuchal and dorsal crests discontinuous, scales of nuchal and dorsal crests enlarged, strongly erected skin fold under nuchal crest and moderately erected skin fold under dorsal crest in males in life, weakly erected skin fold under nuchal crest and no skin fold under dorsal crest in females in life; (9) distinct transverse gular fold present; (10) ventral scales of head heterogeneous in size, anterior and middle ones larger, posterior and side ones smaller, all strongly keeled; (11) ventral scales of body strongly keeled; (12) gular spot absent in both sexes; (13) dorsolateral stripes distinct in males, strongly jagged, pale yellow in life; (14) a series of dark, hollow, approximately rhomboid patterns between dorsolateral stripes on dorsum; (15) a distinct wide black stripe on shoulder fold region on each side; (16) stripes around eye absent or very indistinct; (17) oral cavity, inner lips, and tongue pale flesh colour in life.

#### Description of holotype.

Adult male, SVL 77.0 mm; tail short, TAL 130.0 mm, TAL/SVL 1.69; limbs moderately long, FLL 33.1 mm on left side, FLL/SVL 0.43, HLL 53.5 mm on left side, HLL/SVL 0.69. Head relatively long, HW/HL 0.67, HD/HW 0.84; snout moderately long, SEL/HL 0.37. Rostral elongated, bordered by five small postrostral scales; dorsal head scales heterogeneous, all strongly keeled; distinct Y-shaped ridge on dorsal snout. Nasal oval, separated from first supralabial by two rows of scales on each side; loreals small, keeled; suborbital scale rows 5/4, keeled; canthus rostralis elongated, scales greatly overlapping each other; enlarged, keeled scales forming single lateral ridge from posteroinferior eye to posterosuperior tympanum on each side; tympanum concealed under scales; SL 10/10, smooth. Mental pentagonal; IL 11/11; enlarged chin shields 10/10, smooth, first one connected IL on each side, second one separated from IL by one row of small scales on each side, remaining ones separated from IL by two rows of small scales on each side; ventral head scales heterogeneous in size, anterior and middle ones larger, posterior and lateral ones smaller, all strongly keeled; distinct transverse gular fold present; gular pouch moderately developed.

Distinct shoulder fold present; dorsal body scales heterogeneous in size and shape, all keeled, tip pointing backwards; axillary scales much smaller than remaining dorsals; enlarged dorsal scales forming one intermittent longitudinal row between dorsal crest and dorsolateral stripe on each side, remaining enlarged dorsal scales irregularly scattered on each side of body. Nuchal and dorsal crests discontinuous, separated by a diastema, scales of nuchal and dorsal crests enlarged; strongly erected skin fold under nuchal crest and moderately erected skin fold under dorsal crest; MD 48. Dorsal limb scales strongly keeled, homogeneous on fore-limbs and heterogeneous on hind limbs; F4S 17/16, T4S 21/22. Ventral body scales approximately parallel, homogeneous, all strongly keeled, VN 65. Ventral limb scales parallel, small on upper arms and thighs and larger on forearms and crus, all strongly keeled. Tail scales all strongly keeled, ventral tail scales slightly larger than dorsal tail scales.

#### Colouration of holotype in life.

Dorsal surface of head brownish grey. Two indistinct black transverse bands between orbits on dorsal surface of head. Lateral surfaces of head grey. Two indistinct black stripes from posteroinferior eye to anterior tympanum on each side. Upper lips grey, lower lips white. Oral cavity, inner lips, and tongue with pale flesh colour.

Dorsal surface of body purplish grey. A pale yellow strongly jagged dorsolateral stripe from neck to pelvis on each side of body. A series of dark, hollow, approximately rhomboid-shaped patterns between dorsolateral stripes from neck to base of tail, hollow region pale yellow. A distinct wide black stripe on shoulder fold region on each side. Some irregular black patches below dorsolateral stripe on each side of body, no pale spots on each side of body. Dorsal surfaces of limbs brownish grey with dark transverse bands. Dorsal surface of tail greyish white with distinct dark transverse bands.

Ventral surface of head white with distinct grey reticulated pattern. No gular spot. Ventral surfaces of body and limbs greyish white with no pattern, ventral surface of tail greyish white with indistinct dark transverse bands.

#### Variations.

The variations of metrical characteristics of the type series are provided in Table 2. Other variations are as follows: the transverse bands on the dorsal surface of head and the stripes posteroinferior to the eye are more indistinct in all paratypes; the skin fold under nuchal crest is more weak, no skin fold under dorsal crest, and the dorsolateral stripes are indistinct, pale grey in the female paratype.

**Table 2.** Morphological data of the type series of *Diplodermadanbaense* sp. nov. Morphometric measurements are in the unit of mm. For measurement and count methods and abbreviations, see the Materials and methods.

	KIZ2022048 holotype ♂	KIZ2022049 paratype ♂	KIZ2022050 paratype ♂	KIZ2022051 paratype ♀	KIZ2022056 paratype ♂
SVL	77.0	70.0	68.7	76.6	69.8
TAL	130.0	116.6	122.6	119.1	112.7
HL	26.6	22.7	22.6	23.5	21.4
HW	17.9	16.8	15.0	14.7	16.1
HD	15.0	14.1	12.8	12.8	12.9
SEL	9.9	8.2	8.2	8.4	7.5
FLL	33.1	32.8	32.6	33.6	28.7
HLL	53.5	49.3	48.4	50.0	46.4
T4L	12.2	12.0	12.4	12.1	11.9
TRL	33.2	31.3	29.2	34.5	31.8
TAL/SVL	1.69	1.67	1.78	1.55	1.61
SEL/HL	0.37	0.36	0.36	0.36	0.35
HW/HL	0.67	0.74	0.66	0.63	0.75
HD/HW	0.84	0.84	0.85	0.87	0.80
FLL/SVL	0.43	0.47	0.47	0.44	0.41
HLL/SVL	0.69	0.70	0.70	0.65	0.66
TRL/SVL	0.43	0.45	0.43	0.45	0.46
SL	10/10	9/9	10/9	11/10	10/10
IL	11/11	11/11	11/11	11/11	10/10
NSL	2/2	2/1	1/1	2/2	2/2
MD	48	50	47	58	53
F4S	17/16	16/17	18/18	20/19	19/20
T4S	21/22	22/23	24/23	24/26	25/24
SOR	5/4	4/4	4/5	5/4	5/5
VN	65	72	66	74	68

#### Comparisons.

*Diplodermadanbaense* sp. nov. differs from *D.brevipes* (Gressitt, 1936), *D.chapaense* (Bourret, 1937), *D.fasciatum* (Mertens, 1926), *D.hamptoni* (Smith, 1935), *D.luei* (Ota, Chen & Shang, 1998), *D.makii* (Ota, 1989), *D.menghaiense* Liu, Hou, Wang, Ananjeva & Rao, 2020, *D.micangshanense* (Song, 1987), *D.ngoclinense* (Ananjeva, Orlov & Nguyen, 2017), *D.polygonatum* Hallowell, 1861, *D.swinhonis* (Günther, 1864), and *D.yunnanense* (Anderson, 1878) by the presence of a transverse gular fold (vs. absence).

*Diplodermadanbaense* sp. nov. differs from *D.dymondi* (Boulenger, 1906), *D.panlong* Wang, Che & Siler, 2020, *D.slowinskii* (Rao, Vindum, Ma, Fu & Wilkinson, 2017), *D.varcoae* (Boulenger, 1918), and *D.swild* Wang, Wu, Jiang, Chen, Miao, Siler & Che, 2019 by having concealed tympana (vs. exposed).

*Diplodermadanbaense* sp. nov. differs from *D.angustelinea*, *D.aorun*, *D.batangense* (Li, Deng, Wu & Wang, 2001), *D.bowoense* Wang, Gao, Wu, Siler & Che, 2021, *D.brevicauda* (Manthey, Denzer, Hou & Wang, 2012), *D.daochengense*, *D.flavilabre* Wang, Che & Siler, 2020, *D.formosgulae* Wang, Gao, Wu, Dong, Shi, Qi, Siler & Che, 2021, *D.iadinum* (Wang, Jiang, Siler & Che, 2016), *D.laeviventre* (Wang, Jiang, Siler & Che, 2016), *D.limingensis* Liu, Hou, Rao & Ananjeva, 2022, *D.qilin* Wang, Ren, Che & Siler, 2020, *D.xinlongense* Cai, Zhang, Li, Du, Xie, Hou, Zhou & Jiang, 2022, *D.yangi* Wang, Zhang & Li, 2022, *D.yongshengense*, *D.yulongense*, and *D.zhaoermii* (Gao & Hou, 2002) by the absence of a gular spot in males in life (vs. presence of a colourful gular spot).

*Diplodermadanbaense* sp. nov. differs from *D.drukdaypo* (Wang, Ren, Jiang, Zou, Wu, Che & Siler, 2019) by having strongly keeled ventral scales of body (vs. smooth or weakly keeled); from *D.grahami* (Stejneger, 1924) by having relatively longer hind limbs (HLL/SVL 0.65–0.70 vs. 0.61), having a distinct transverse gular fold (vs. feeble), the absence of a gular spot after preservation (vs. presence), and the presence of dorsolateral stripes (vs. absence); from *D.kangdingense* Cai, Zhang, Li, Du, Xie, Hou, Zhou & Jiang, 2022 by the presence of distinct, dark, hollow, approximately rhomboid patterns between dorsolateral stripes on dorsum (vs. absence) and having greyish white ventrolateral surface of body in males in life (vs. yellow); from *D.panchi* Wang, Zheng, Xie, Che & Siler, 2020 by the presence of distinct, dark, hollow, approximately rhomboid patterns between dorsolateral stripes on dorsum (vs. absence) and the presence of a skin fold under nuchal crest in females in life (vs. absence); from *D.shuoquense* Liu, Hou, Rao & Ananjeva, 2022 by having strongly keeled ventral head scales (vs. smooth or weakly keeled), the absence of distinct radial stripes around the eyes (vs. presence), and the presence of skin folds under nuchal and dorsal crests in males in life (vs. absence); from *D.splendidum* (Barbour & Dunn, 1919) by having strongly jagged dorsolateral stripes in males (vs. smooth); and from *D.vela* (Wang, Jiang & Che, 2015) by having discontinuous nuchal and dorsal crests and skin folds in males in life (vs. continuous nuchal and dorsal crests on continuous skin fold).

*Diplodermadanbaense* sp. nov. is phylogenetically sister to and most similar in morphology characteristic and colouration to *D.flaviceps*; however, *Diplodermadanbaense* sp. nov. can be differentiated from the latter by having a relatively shorter tail (TAL/SVL 1.61–1.78 vs. 1.88–2.09 in males, 1.55 vs. 1.73–2.17 in females), having relatively shorter hind limbs (HLL/SVL 0.66–0.70 vs. 0.72–0.80 in males, 0.65 vs. 0.70–0.81 in females), having a smaller ratio of head width to head length (HW/HL 0.66–0.75 vs. 0.76–0.84 in males, 0.63 vs. 0.71–0.78 in females), having a greater ratio of head depth to head width (HD/HW 0.80–0.85 vs. 0.70–0.78 in males, 0.87 vs. 0.75–0.83 in females), having relatively shorter fourth toes (T4L/SVL 0.16–0.18 vs. 0.18–0.21 in males, 0.16 vs. 0.17–0.21 in females), and having a moderately erected skin fold under dorsal crest in males in life (vs. strongly erected) and the absence of a skin fold under dorsal crest in females in life (vs. presence).

**Figure 4.**
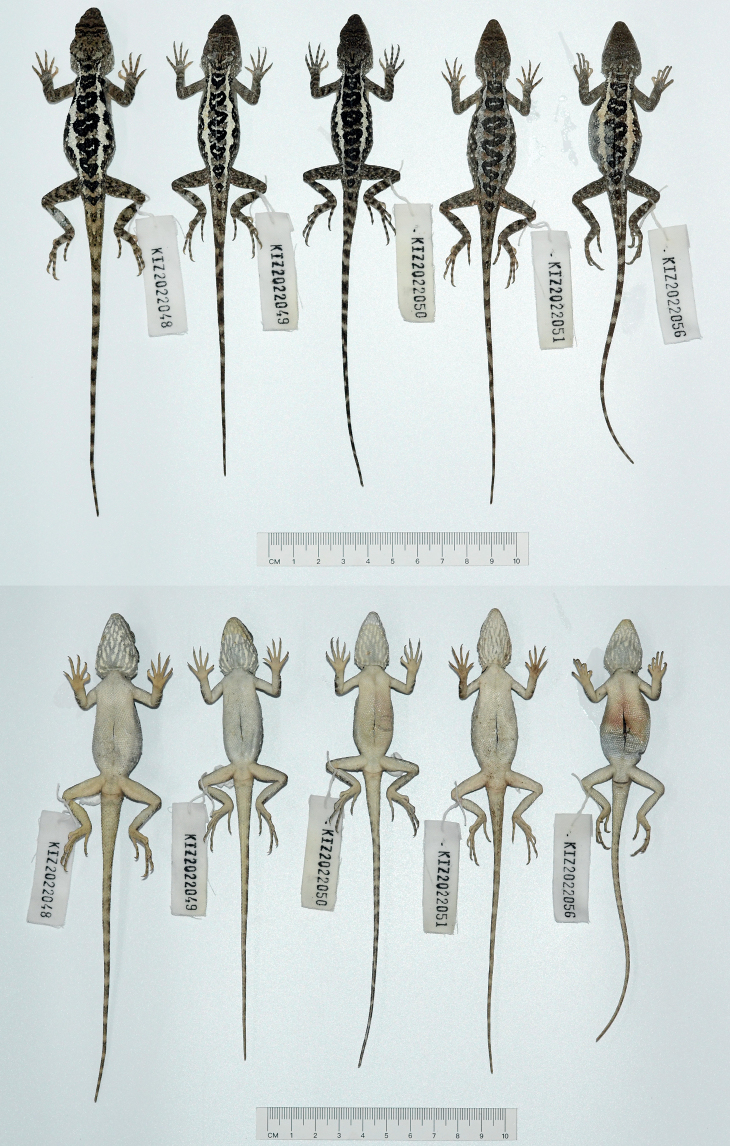
Dorsal view (upper) and ventral view (lower) of the type series of *Diplodermadanbaense* sp. nov. in preservative.

**Figure 5.**
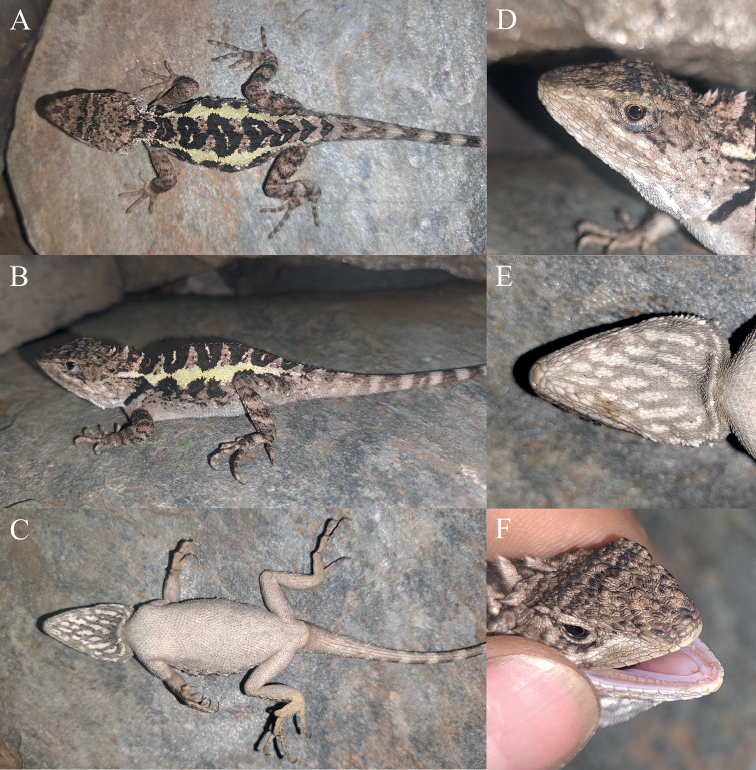
Holotype (KIZ2022048) of *Diplodermadanbaense* sp. nov. in life **A** dorsal view **B** lateral view **C** ventral view **D** close-up view of the dorsolateral side of the head **E** close-up view of the ventral side of the head **F** close-up view of the oral cavity.

**Figure 6.**
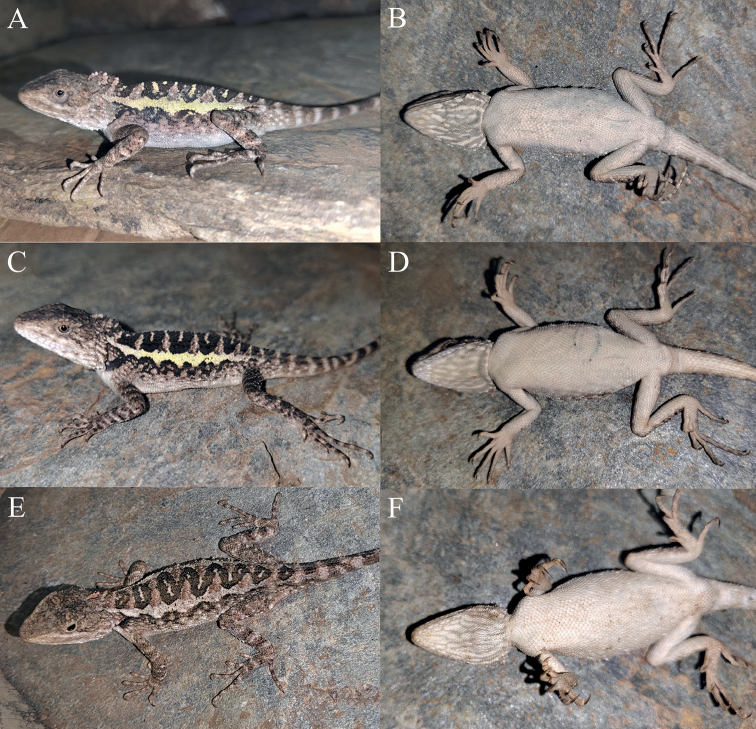
Paratypes of *Diplodermadanbaense* sp. nov. in life **A, B** the male paratype KIZ2022049 **C, D** the male paratype KIZ2022050 **E, F** the female paratype KIZ2022051

#### Distribution.

This species is currently known only from its type locality in Danba County, Ganzi Prefecture, Sichuan Province, China (Fig. 1).

#### Natural history.

This species is terrestrial, inhabiting the hot-dry valley of the upper Dadu River. There are a few trees and many rocks at the type locality (Fig. 16A, B). All specimens were collected between 11 and 12 a.m. when they were basking on rock piles.

### ﻿ Diploderma donglangense sp. nov.

Taxon classificationAnimaliaSquamataAgamidae

31C970F2-58ED-5636-802D-5537A97ABD1C

https://zoobank.org/D964D8B1-6004-457A-95E0-0B6EAA97B7E0

Figs 7 , 8 , 9

#### Type material.

***Holotype*.** KIZ2022057, adult male, collected on 31 July 2022 by Mian Hou from Yaying Village, Donglang Township, Muli County, Liangshan Prefecture, Sichuan Province, China (28°48'49"N, 100°35'42"E, 2750 m elevation).

***Paratypes*.** KIZ2022058–KIZ2022059, KIZ2022061, three adult females; KIZ2022060, adult male; collecting information all the same as the holotype.

#### Etymology.

The specific epithet refers to Donglang Township, where the new species was discovered.

#### Diagnosis.

*Diplodermadonglangense* sp. nov. can be diagnosed from congeners by a combination of the following morphological characteristics: (1) body size relatively small, SVL 44.9–52.8 mm in adult males, 47.2–56.4 in adult females; (2) tail relatively short, TAL/SVL 1.76–1.87 in adult males, 1.59–1.89 in adult females; (3) limbs moderately long, FLL/SVL 0.46–0.47 in adult males, 0.44–0.50 in adult females, HLL/SVL 0.73–0.75 in adult males, 0.70–0.78 in adult females; (4) head moderately long, HW/HL 0.69–0.71 in adult males, 0.71–0.73 in adult females; (5) MD 37–43; (6) F4S 13–17, T4S 19–24; (7) tympanum concealed; (8) nuchal and dorsal crest scales feebly developed, no skin fold under nuchal and dorsal crests; (9) distinct transverse gular fold present; (10) ventral scales of head homogeneous in size, keeled; (11) ventral scales of body strongly keeled; (12) gular spot present in males, present or absent in females, pale yellow in life; (13) dorsolateral stripes distinct in males, moderately jagged, creamy yellow in life; (14) radial stripes around eye indistinct; (15) oral cavity, inner lips, and tongue pale pink in life.

#### Description of holotype.

Adult male, SVL 52.8 mm; tail relatively short, TAL 93.1 mm, TAL/SVL 1.76; limbs moderately long, FLL 24.4 mm on left side, FLL/SVL 0.46, HLL 38.8 mm on left side, HLL/SVL 0.73. Head moderately long, HW/HL 0.69, HD/HW 0.79; snout relatively short, SEL/HL 0.33. Rostral rectangular, bordered by six small postrostral scales; dorsal head scales heterogeneous, all strongly keeled; distinct Y-shaped ridge on dorsal snout. Nasal oval, separated from first supralabial by two rows of scales on each side; loreals small, keeled; suborbital scale rows 5/4, keeled; canthus rostralis elongated, greatly overlapping with each other; enlarged, keeled scales forming distinct single lateral ridge from posteroinferior eye to posterosuperior tympanum on each side; tympanum concealed under scales; SL 10/9, feebly keeled. Mental pentagonal; IL 11/11; enlarged chin shields 4/4, smooth, first one contacting IL on each side, remaining ones separated from IL by one or two rows of small scales on each side; ventral head scales homogeneous in size, keeled; distinct transverse gular fold present; gular pouch weakly developed.

Distinct shoulder fold present; dorsal body scales heterogeneous in size and shape, all keeled, tip pointing backwards; axillary scales much smaller than remaining dorsals; enlarged dorsal scales forming one continuous longitudinal row between dorsal crest and dorsolateral stripe on each side, remaining enlarged dorsal scales roughly forming three or four longitudinal rows on each side of body. Nuchal and dorsal crests feebly developed; no skin fold under nuchal and dorsal crests; MD 40. Dorsal limb scales strongly keeled, mostly homogeneous, except a few enlarged, conical scales on postaxial thighs; F4S 16/16, T4S 24/23. Ventral body scales approximately parallel, homogeneous, all strongly keeled, VN 63. Ventral limb scales parallel, homogeneous, approximately equal in size to ventrals, all strongly keeled. Tail scales all strongly keeled, ventral tail scales larger than dorsal tail scales.

#### Colouration of holotype in life.

Dorsal surface of head dark brown. Transverse bands on dorsal surface of head indistinct. Lateral surfaces of head brownish grey. A distinct black stripe from posteroinferior eye to tympanum region on each side. Upper lips brownish grey, lower lips white. Oral cavity, inner lips, and tongue pale pink.

Dorsal surface of body dark brown. A creamy yellow moderately jagged dorsolateral stripe on each side of body from occipital region to pelvis. Some indistinct black patterns between two dorsolateral stripes. Some creamy yellow spots scattered below dorsolateral stripe on each side of body. Dorsal surfaces of limbs brown with dark transverse bands. Dorsal surface of tail brownish grey with indistinct dark transverse bands.

Ventral surface of head white. A pale yellow gular spot on posterior central part of ventral head, some short black stripes mostly on anterior and sides of gular spot. Ventral surfaces of body, limbs, and tail white with no pattern.

#### Variations.

The variations of metrical characteristics of the type series are provided in Table 3. Other variations are as follows: the dorsal colour are paler, the patterns between two dorsolateral stripes are more distinct, the transverse bands on the dorsal surface of head are more distinct, and the dorsolateral stripes are indistinct, pale grey or yellow anteriorly and pale grey posteriorly in the female paratypes; the gular spot present in one female paratype and absent in another female paratype; in addition, the short black stripes on ventral surface of head are more indistinct in all paratypes.

**Table 3.** Morphological data of the type series of *Diplodermadonglangense* sp. nov. Morphometric measurements are in the unit of mm. For measurement and count methods and abbreviations, see the Materials and methods.

	KIZ2022057 holotype ♂	KIZ2022058 paratype ♀	KIZ2022059 paratype ♀	KIZ2022060 paratype ♂	KIZ2022061 paratype ♀
SVL	52.8	51.2	56.4	44.9	47.2
TAL	93.1	81.4	90.5	84.0	89.1
HL	16.9	16.1	17.3	14.6	15.0
HW	11.7	11.8	12.2	10.4	10.7
HD	9.3	9.6	9.7	8.4	8.5
SEL	5.6	5.5	6.1	5.3	5.3
FLL	24.4	24.1	24.6	21.2	23.8
HLL	38.8	40.1	39.2	33.6	35.4
T4L	9.0	9.2	9.4	8.0	9.5
TRL	23.5	23.0	25.9	19.8	20.8
TAL/SVL	1.76	1.59	1.60	1.87	1.89
SEL/HL	0.33	0.34	0.35	0.36	0.35
HW/HL	0.69	0.73	0.71	0.71	0.71
HD/HW	0.79	0.81	0.80	0.81	0.79
FLL/SVL	0.46	0.47	0.44	0.47	0.50
HLL/SVL	0.73	0.78	0.70	0.75	0.75
TRL/SVL	0.45	0.45	0.46	0.44	0.44
SL	10/9	9/9	8/9	8/8	10/10
IL	11/11	9/10	10/10	10/9	10/9
NSL	2/2	2/2	2/1	1/1	2/1
MD	40	37	43	39	41
F4S	16/16	13/14	17/17	14/15	16/15
T4S	24/23	19/20	21/21	22/22	24/21
SOR	5/4	5/5	4/4	5/5	5/5
VN	63	56	60	61	55

#### Comparisons.

*Diplodermadonglangense* sp. nov. differs from *D.brevipes*, *D.chapaense*, *D.fasciatum*, *D.hamptoni*, *D.luei*, *D.makii*, *D.menghaiense*, *D.micangshanense*, *D.ngoclinense*, *D.polygonatum*, *D.swinhonis*, and *D.yunnanense* by the presence of a transverse gular fold (vs. absence).

*Diplodermadonglangense* sp. nov. differs from *D.dymondi*, *D.panlong*, *D.slowinskii*, *D.varcoae*, and *D.swild* by having concealed tympana (vs. exposed).

*Diplodermadonglangense* sp. nov. differs from *D.drukdaypo*, *D.flaviceps*, *D.shuoquense*, *D.splendidum*, and *D.vela* by the presence of a distinct gular spot in males in life (vs. absence).

*Diplodermadonglangense* sp. nov. differs from *D.aorun*, *D.batangense*, *D.bowoense*, *D.brevicauda*, *D.daochengense*, *D.flavilabre*, *D.formosgulae*, *D.iadinum*, *D.laeviventre*, *D.limingensis*, *D.xinlongense*, *D.yangi*, *D.yongshengense*, *D.yulongense*, and *D.zhaoermii* by having a pale yellow gular spot in males in life (vs. chartreuse, blue, green, lilac, orange, or yellowish white).

*Diplodermadonglangense* sp. nov. differs from *D.angustelinea* by the presence of short black stripes on ventral head (vs. absence); from *D.grahami* by having relatively longer hind limbs (HLL/SVL 0.70–0.78 vs. 0.61), having a distinct transverse gular fold (vs. feeble), and the presence of dorsolateral stripes (vs. absence); from *D.kangdingense* by the absence of skin folds under nuchal and dorsal crests in males (vs. presence) and having white ventrolateral surface of body in males in life (vs. yellow); from *D.panchi* by having a relatively longer tail in females (TAL/SVL 1.59–1.89 vs. 1.42–1.52), having relatively longer hind limbs in females (HLL/SVL 0.70–0.78 vs. 0.60–0.66), and the presence of short black stripes on ventral head (vs. absence); and from *D.qilin* by having short black stripes on ventral head (vs. vermiculate stripes) and having moderately jagged dorsolateral stripes in males (vs. strongly jagged).

*Diplodermadonglangense* sp. nov. differs from *Diplodermadanbaense* sp. nov. by the presence of a distinct gular spot in males in life (vs. absence), the absence of reticulate pattern on ventral head (vs. presence), having moderately jagged dorsolateral stripes in males (vs. strongly jagged), the absence of distinct, dark, hollow, approximately rhomboid patterns between dorsolateral stripes on dorsum (vs. absence), and the absence of skin folds under nuchal and dorsal crests in males (vs. presence).

**Figure 7.**
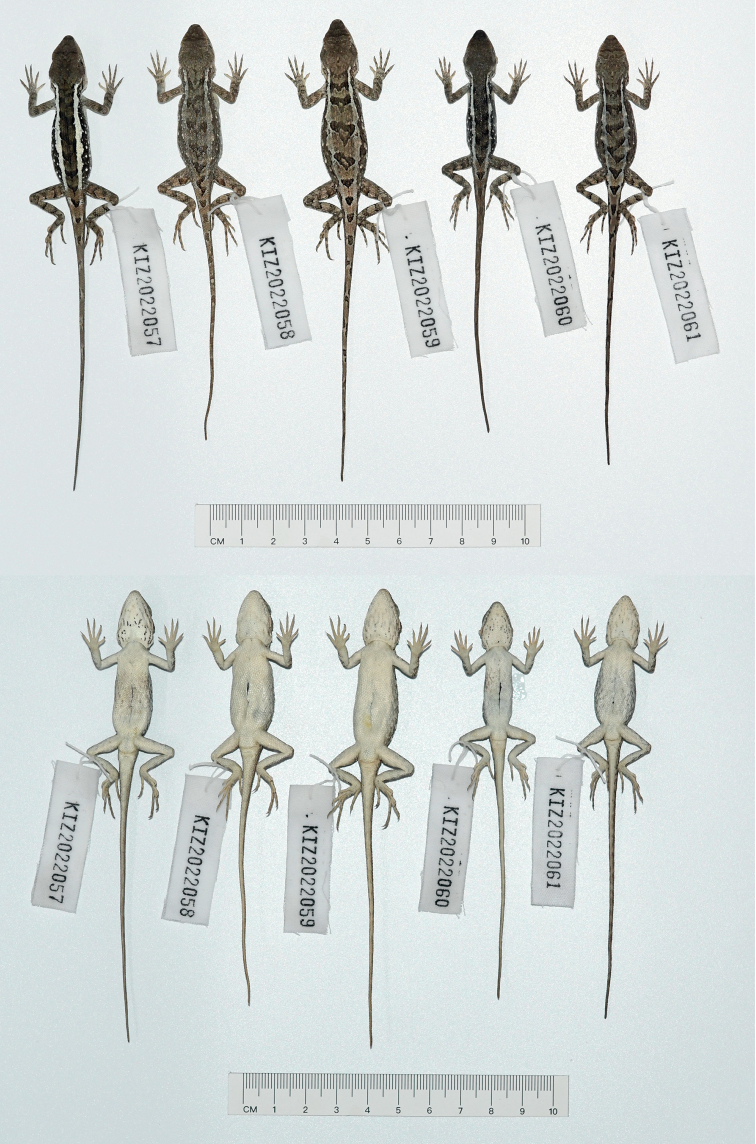
Dorsal view (upper) and ventral view (lower) of the type series of *Diplodermadonglangense* sp. nov. in preservative.

**Figure 8.**
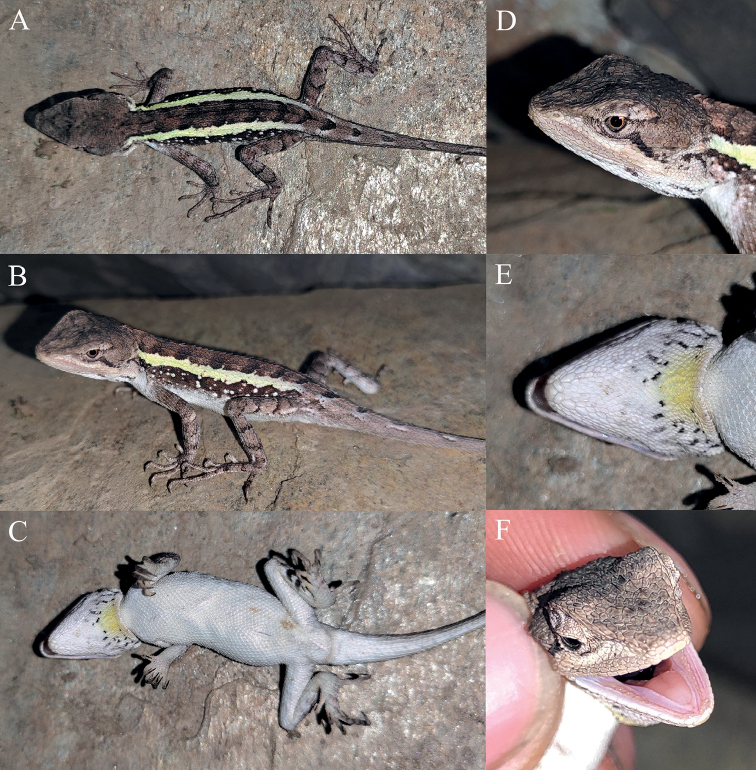
Holotype (KIZ2022057) of *Diplodermadonglangense* sp. nov. in life **A** dorsal view **B** lateral view **C** ventral view **D** close-up view of the dorsolateral side of the head **E** close-up view of the ventral side of the head **F** close-up view of the oral cavity.

**Figure 9.**
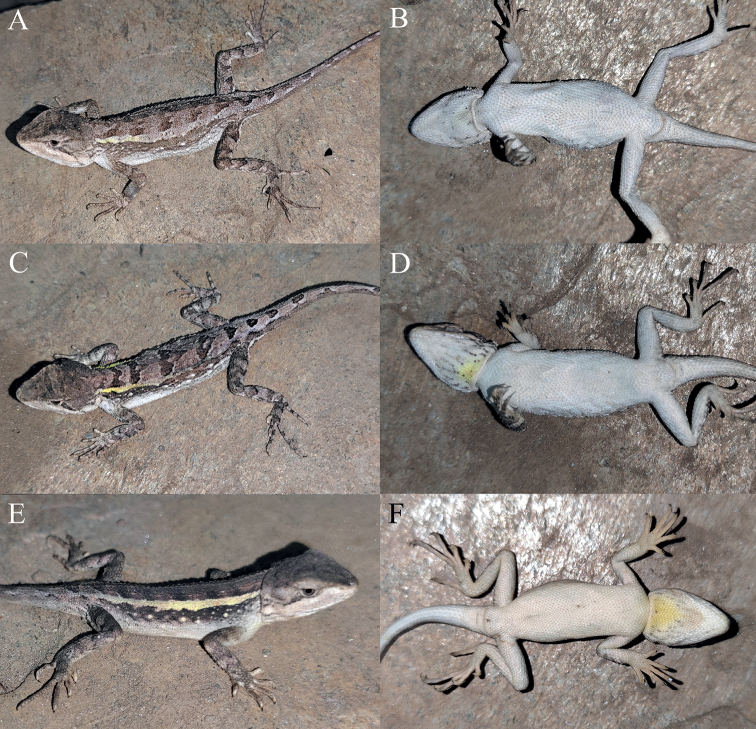
Paratypes of *Diplodermadonglangense* sp. nov. in life **A, B** the female paratype KIZ2022058 **C, D** the female paratype KIZ2022059 **E, F** dorsolateral the male paratype KIZ2022060.

#### Distribution.

This species is currently known only from its type locality in Donglang Township, Muli County, Liangshan Prefecture, Sichuan Province, China (Fig. 1).

#### Natural history.

This species is terrestrial, inhabiting the hot-dry valley of the upper Shuiluo River, which is a tributary of the Jinsha River. There are many thorny shrubs and some rock piles at the type locality (Fig. 16C, D). All specimens were collected between 1 and 3 p.m. when they were basking on rock piles.

### ﻿ Diploderma jiulongense sp. nov.

Taxon classificationAnimaliaSquamataAgamidae

DB30AD3D-1134-5420-B72B-60E42ECAEABA

https://zoobank.org/A60F9BDB-0363-41A2-A9B9-CE7F6E964B56

Figs 10 , 11 , 12

#### Type material.

***Holotype*.** KIZ2022086, adult male, collected on 8 August 2022 by Mian Hou from Yandai Town, Jiulong County, Ganzi Prefecture, Sichuan Province, China (28°28'39"N, 101°44'57"E, 1680 m elevation).

***Paratypes*.** KIZ2022087 and KIZ2022101, two adult females; KIZ2022099–KIZ2022100, two adult males, collecting information all the same as the holotype.

#### Etymology.

The specific epithet refers to Jiulong County, where the new species was discovered.

#### Diagnosis.

*Diplodermajiulongense* sp. nov. can be diagnosed from congeners by a combination of the following morphological characteristics: (1) body size moderate, SVL 44.5–51.4 mm in adult males, 61.7–64.4 in adult females; (2) tail very long, TAL/SVL 2.57–2.71 in adult males, 2.33–2.44 in adult females; (3) limbs very long, FLL/SVL 0.50–0.53 in adult males, 0.46–0.47 in adult females, HLL/SVL 0.86–0.94 in adult males, 0.79–0.82 in adult females; (4) head moderately long, HW/HL 0.68–0.73 in adult males, 0.71–0.72 in adult females; (5) MD 36–40; (6) F4S 16–19, T4S 23–27; (7) tympanum concealed; (8) nuchal and dorsal crests continuous, feebly developed, no skin fold under nuchal and dorsal crests; (9) distinct transverse gular fold present; (10) ventral scales of head heterogeneous in size, ones on centre of gular pouch largest, all strongly keeled; (11) ventral scales of body strongly keeled; (12) gular spot present in males, indistinct or absent in females, pale yellow in life; (13) dorsolateral stripes distinct in males, narrow and smooth edged, bright yellow in life; (14) no radial stripes around eye; (15) oral cavity and inner lips pinkish white, tongue pale flesh colour in life.

#### Description of holotype.

Adult male, SVL 51.4 mm; tail very long, TAL 137.1 mm, TAL/SVL 2.67; limbs very long, FLL 27.0 mm on left side, FLL/SVL 0.53, HLL 48.1 mm on left, HLL/SVL 0.94. Head moderately long, HW/HL 0.68, HD/HW 0.86; snout moderately long, SEL/HL 0.37. Rostral elongated, bordered by six small postrostral scales; dorsal head scales heterogeneous, all strongly keeled; distinct Y-shaped ridge on dorsal snout. Nasal oval, separated from first supralabial by single row of scales on each side; loreals small, keeled; suborbital scale rows 4/4, keeled; canthus rostralis elongated, greatly overlapping with each other; enlarged, keeled scales forming single lateral ridge from posteroinferior eye to posterosuperior tympanum on each side; tympanum concealed under scales; SL 8/8, smooth. Mental pentagonal; IL 9/9; enlarged chin shields 4/5, smooth, first one contacting IL on each side, remaining ones separated from IL by two rows of small scales on each side; ventral head scales heterogeneous in size, ones on centre of gular pouch largest, all strongly keeled; distinct transverse gular fold present; gular pouch weakly developed.

Distinct shoulder fold present; dorsal body scales heterogeneous in size and shape, all keeled, tip pointing backwards; axillary scales much smaller than remaining dorsals; enlarged dorsal scales roughly forming four longitudinal rows on each side of body. Nuchal and dorsal crests feebly developed, continuous; no skin fold under nuchal and dorsal crest; MD 36. Dorsal limb scales strongly keeled, mostly homogeneous, except a few enlarged, conical scales on postaxial thighs; F4S 18/19, T4S 27/27. Ventral body scales approximately parallel, homogeneous, all strongly keeled, VN 54. Ventral limb scales parallel, small on upper arms and thighs and larger on forearms and crus, all strongly keeled. Tail scales all strongly keeled, ventral tail scales slightly larger than dorsal tail scales.

#### Colouration of holotype in life.

Dorsal surface of head dark grey with no transverse bands. Lateral surfaces of head grey, a greyish white suborbital stripe extending from nasal scale to rictus on each side. Upper lips brownish grey, lower lips greyish white. No radial stripes around eyes. Oral cavity and inner lips pinkish white, tongue pale flesh colour.

Dorsal surface of body brownish black. A narrow, bright yellow, smooth-edged, dorsolateral stripe on each side of body from occipital region to pelvis. Some black inverted triangular patterns between the two dorsolateral stripes. Some bright yellow spots scattered below dorsolateral stripe on each side of body. Dorsal surfaces of limbs dark grey. Dark transverse bands on dorsal surfaces of limbs very indistinct. Dorsal surface of tail brownish grey with some indistinct dark transverse bands.

Ventral surface of head greyish white. A pale yellow gular spot present on posterior central part of ventral head, no stripes on ventral head. Ventral surfaces of body, limbs, and tail greyish white with no pattern.

#### Variations.

The variations of metrical characteristics of the type series are provided in Table 4. Other variations are as follows: the dorsal colour is brownish red or brick red, the transverse bands on the limbs are more distinct, the dorsolateral stripes are indistinct, pale grey or yellow anteriorly and pale grey posteriorly, and the gular spot indistinct or absent in the female paratypes.

**Table 4.** Morphological data of the type series of *Diplodermajiulongense* sp. nov. Morphometric measurements are in the unit of mm. For measurement and count methods and abbreviations, see the Materials and methods.

	KIZ2022086 holotype ♂	KIZ2022087 paratype ♀	KIZ2022099 paratype ♂	KIZ2022100 paratype ♂	KIZ2022101 paratype ♀
SVL	51.4	61.7	47.8	44.5	64.4
TAL	137.1	143.5	129.7	114.3	157.4
HL	17.4	18.0	14.5	13.4	18.5
HW	11.9	13.0	10.6	9.5	13.1
HD	10.2	10.5	8.6	7.9	10.3
SEL	6.4	6.5	5.6	4.6	7.0
FLL	27.0	28.7	24.9	22.3	29.8
HLL	48.1	50.6	40.9	38.2	51.1
T4L	12.8	12.9	10.1	10.0	12.6
TRL	21.9	31.0	21.6	20.6	31.2
TAL/SVL	2.67	2.33	2.71	2.57	2.44
SEL/HL	0.37	0.36	0.39	0.34	0.38
HW/HL	0.68	0.72	0.73	0.71	0.71
HD/HW	0.86	0.86	0.81	0.83	0.79
FLL/SVL	0.53	0.47	0.52	0.50	0.46
HLL/SVL	0.94	0.82	0.86	0.86	0.79
TRL/SVL	0.43	0.50	0.45	0.46	0.48
SL	8/8	8/8	8/8	9/9	8/9
IL	9/9	9/9	10/10	9/8	10/9
NSL	1/1	1/1	1/1	1/1	2/2
MD	36	40	36	37	36
F4S	18/19	17/16	16/16	16/18	17/17
T4S	27/27	25/25	23/23	23/-	24/23
SOR	4/4	3/4	4/4	3/4	3/3
VN	54	52	54	56	55

#### Comparisons.

*Diplodermajiulongense* sp. nov. differs from *D.brevipes*, *D.chapaense*, *D.fasciatum*, *D.hamptoni*, *D.luei*, *D.makii*, *D.menghaiense*, *D.micangshanense*, *D.ngoclinense*, *D.polygonatum*, *D.swinhonis*, and *D.yunnanense* by the presence of a transverse gular fold (vs. absence).

*Diplodermajiulongense* sp. nov. differs from *D.dymondi*, *D.panlong*, *D.slowinskii*, *D.varcoae*, and *D.swild* by having concealed tympana (vs. exposed).

*Diplodermajiulongense* sp. nov. differs from *D.drukdaypo*, *D.flaviceps*, *D.shuoquense*, *D.splendidum*, and *D.vela* by the presence of a distinct gular spot in males in life (vs. absence).

*Diplodermajiulongense* sp. nov. differs from *D.aorun*, *D.batangense*, *D.bowoense*, *D.brevicauda*, *D.daochengense*, *D.flavilabre*, *D.formosgulae*, *D.iadinum*, *D.laeviventre*, *D.limingensis*, *D.xinlongense*, *D.yangi*, *D.yongshengense*, *D.yulongense*, and *D.zhaoermii* by having a pale yellow gular spot in males in life (vs. chartreuse, blue, green, lilac, orange, or yellowish white).

*Diplodermajiulongense* sp. nov. differs from *D.grahami* by having a relatively much longer tail (TAL/SVL 2.33–2.71 vs. 1.64), having relatively longer hind limbs (HLL/SVL 0.82–0.94 vs. 0.61), having a distinct transverse gular fold (vs. feeble), and the presence of dorsolateral stripes (vs. absence); from *D.kangdingense* by having a relatively longer tail (TAL/SVL 2.33–2.71 vs. 1.56–2.27), the absence of skin folds under nuchal and dorsal crests in males (vs. presence), and having greyish white ventrolateral surface of body in males in life (vs. yellow); from *D.panchi* by having a relatively longer tail in females (TAL/SVL 2.33–2.44 vs. 1.42–1.52), having relatively longer hind limbs in females (HLL/SVL 0.79–0.82 vs. 0.60–0.66), and the presence of short black stripes on ventral head (vs. absence); and from *D.qilin* by having a relatively longer tail (TAL/SVL 2.33–2.71 vs. 1.74–2.18), having short black stripes on ventral head (vs. vermiculate stripes) and having smooth edged dorsolateral stripes in males (vs. strongly jagged).

*Diplodermajiulongense* sp. nov. is phylogenetically sister to and most similar in morphology characteristic and colouration to *D.angustelinea*; however, *Diplodermajiulongense* sp. nov. can be differentiated from the latter by having a relatively longer tail (TAL/SVL 2.57–2.71 vs. 2.30–2.49 in males, 2.33–2.44 vs. 1.94–2.22 in females), having relatively longer fore-limbs (FLL/SVL 0.50–0.53 vs. 0.46–0.47 in males, 0.46–0.47 vs. 0.41–0.46 in females), having relatively longer hind limbs (HLL/SVL 0.86–0.94 vs. 0.71–0.80 in males, 0.79–0.82 vs. 0.74–0.79 in females), having a greater ratio of head depth to head width (HD/HW 0.81–0.86 vs. 0.73–0.79 in males, 0.79–0.86 vs. 0.73–0.79 in females), and having smooth edged dorsolateral stripes in males (vs. weakly jagged).

*Diplodermajiulongense* sp. nov. differs from *Diplodermadanbaense* sp. nov. by having a relatively longer tail (TAL/SVL 2.33–2.71 vs. 1.55–1.78), the presence of a distinct gular spot in males in life (vs. absence), the absence of reticulate pattern on ventral head (vs. presence), having smooth edged dorsolateral stripes in males (vs. strongly jagged), the absence of distinct, dark, hollow, approximately rhomboid patterns between dorsolateral stripes on dorsum (vs. absence), and the absence of skin folds under nuchal and dorsal crests in males (vs. presence).

*Diplodermajiulongense* sp. nov. differs from *Diplodermadonglangense* sp. nov. by having a relatively longer tail (TAL/SVL 2.33–2.71 vs. 1.59–1.89), the absence of short black stripes on ventral head (vs. presence), and having smooth edged dorsolateral stripes in males (vs. moderately jagged).

**Figure 10.**
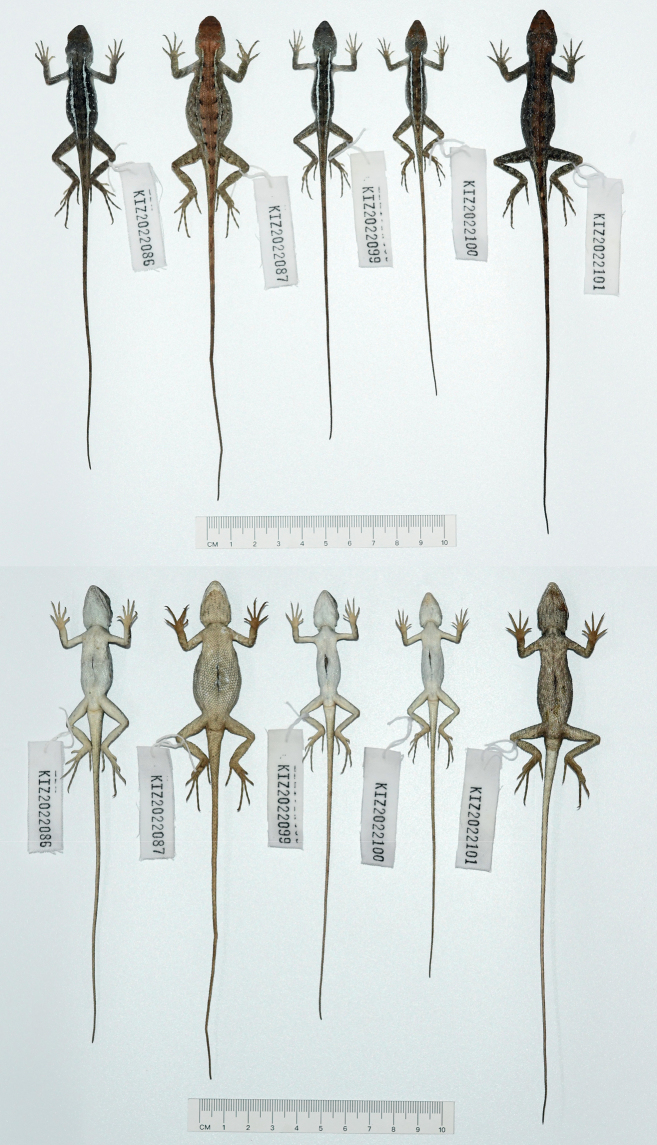
Dorsal view (upper) and ventral view (lower) of the type series of *Diplodermajiulongense* sp. nov. in preservative.

**Figure 11.**
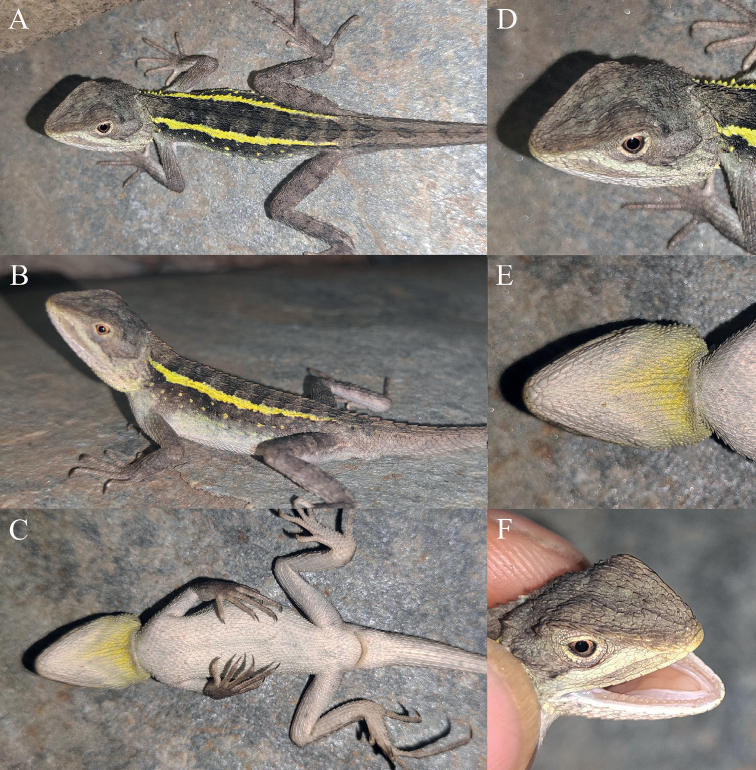
Holotype (KIZ2022086) of *Diplodermajiulongense* sp. nov. in life **A** dorsal view **B** lateral view **C** ventral view **D** close-up view of the dorsolateral side of the head **E** close-up view of the ventral side of the head **F** close-up view of the oral cavity.

**Figure 12.**
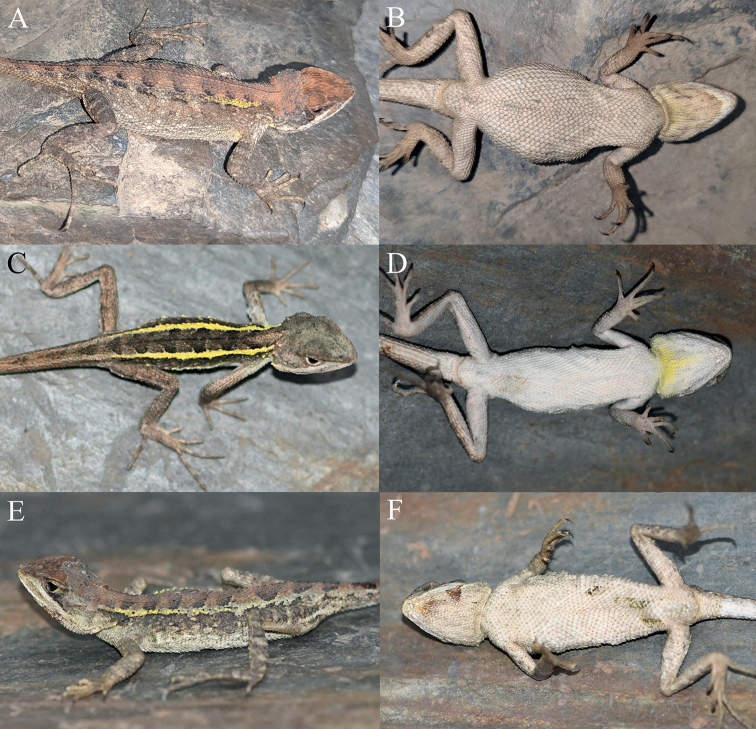
Paratypes of *Diplodermajiulongense* sp. nov. in life **A, B** the female paratype KIZ2022087 **C, D** the male paratype KIZ2022099 **E, F** the female paratype KIZ2022101.

#### Distribution.

This species is currently known only from its type locality in Yandai Town, Jiulong County, Ganzi Prefecture, Sichuan Province, China (Fig. 1).

#### Natural history.

All specimens were collected between 9 and 12 a.m. in bushes or grass in the Yalong River valley. There are a few trees and many rocks at the type locality (Fig. 16E, F).

### ﻿ Diploderma tachengense sp. nov.

Taxon classificationAnimaliaSquamataAgamidae

959E4228-0C76-54E2-B31F-29E51CC2D313

https://zoobank.org/9FAC8DF8-7A21-48C7-B6E3-3463B16347B6

Figs 13 , 14 , 15

#### Type material.

***Holotype*.** KIZ2022028, adult male, collected on 6 July 2022 by Shuo Liu from Kena Village, Tacheng Town, Weixi County, Diqing Prefecture, Yunnan Province, China (27°34'12"N, 99°20'36"E, 2180 m elevation).

***Paratypes*.** KIZ2022027, adult male; KIZ2022029, adult female; KIZ2022038, subadult male; and KIZ2022039, subadult female; collecting information all the same as the holotype.

#### Etymology.

The specific epithet refers to Tacheng Town, where the new species was discovered.

#### Diagnosis.

*Diplodermatachengense* sp. nov. can be diagnosed from congeners by a combination of the following morphological characteristics: (1) body size moderate, SVL 55.2–55.7 mm in adult males, 65.4 mm in adult female; (2) tail moderately long, TAL/SVL 1.88–1.89 in adult males, 1.56 in adult female; (3) limbs moderately long, FLL/SVL 0.45–0.47 in adult males, 0.46 in adult female, HLL/SVL 0.76–0.78 in adult males, 0.70 in adult female; (4) head relatively robust, HW/HL 0.73–0.80 in adult males, 0.74 in adult female; (5) MD 38–44; (6) F4S 15–19, T4S 20–24; (7) tympanum concealed; (8) nuchal and dorsal crests continuous, feebly developed, no skin fold under nuchal and dorsal crests; (9) distinct transverse gular fold present; (10) ventral scales of head homogeneous, feebly keeled; (11) ventral scales of body strongly keeled; (12) gular spot present in both sexes, pale yellow in life; (13) dorsolateral stripes distinct in males, strongly jagged, pale yellow in life; (14) radial stripes around eye relatively distinct; (15) oral cavity and inner lips pinkish white, tongue pink in life.

#### Description of holotype.

Adult male, SVL 55.2 mm; tail moderately long, TAL 104.5 mm, TAL/SVL 1.89; limbs moderately long, FLL 25.9 mm on left side, FLL/SVL 0.47, HLL 41.7 mm on left, HLL/SVL 0.76. Head relatively robust, HW/HL 0.73, HD/HW 0.87; snout moderately long, SEL/HL 0.36. Rostral elongated, bordered by six small postrostral scales; dorsal head scales heterogeneous, all strongly keeled; distinct Y-shaped ridge on dorsal snout. Nasal oval, separated from first supralabial by single row of scales on each side; loreals small, keeled; suborbital scale rows 4/4, keeled; canthus rostralis elongated, greatly overlapping with each other; enlarged, keeled scales forming distinct single lateral ridge from posteroinferior eye to posterosuperior tympanum on each side; tympanum concealed under scales; SL 9/9, feebly keeled. Mental approximately triangular; IL 10/10; enlarged chin shields 4/4, smooth, first one contacting IL on each side, second ones separated from IL by one row of small scales on each side, remaining ones separated from IL by two rows of small scales on each side; ventral head scales homogeneous in size, feebly keeled; distinct transverse gular fold present; gular pouch weakly developed.

Distinct shoulder fold present; dorsal body scales heterogeneous in size and shape, all keeled, tip pointing backwards; axillary scales much smaller than remaining dorsals; enlarged dorsal scales forming one intermittent longitudinal row between dorsal crest and dorsolateral stripe on each side, remaining enlarged dorsal scales irregularly scattered on each side of body. Nuchal and dorsal crests feebly developed, continuous; no skin fold under nuchal and dorsal crests; MD 43. Dorsal limb scales strongly keeled, mostly homogeneous, except a few enlarged, conical scales on postaxial thighs; F4S 16/17, T4S 21/22. Ventral body scales approximately parallel, homogeneous, all strongly keeled, VN 55. Ventral limb scales parallel, small on upper arms and thighs and larger on forearms and crus, all strongly keeled. Tail scales all strongly keeled, ventral tail scales slightly larger than dorsal tail scales.

#### Colouration of holotype in life.

Dorsal surface of head dark brown with indistinct transverse bands. Lateral surfaces of head brownish yellow. Upper lips brownish grey, lower lips white. Distinct radial stripes around eye on each side. Oral cavity and inner lips pinkish white, tongue pink.

Dorsal surface of body brownish black. A pale yellow strongly jagged dorsolateral stripe on each side of body from occipital region to pelvis. Several pale yellow spots scattered below dorsolateral stripe on each side of body. Dorsal surfaces of limbs brown. Distinct dark transverse bands present on dorsal surfaces of limbs. Dorsal surface of tail brown with indistinct dark transverse bands.

Ventral surface of head white. A pale yellow gular spot present on posterior central part of ventral head, indistinct dark stripes on other parts of ventral head. Ventral surface of body white with no pattern, ventral surfaces of limbs and tail reddish white.

**Figure 13.**
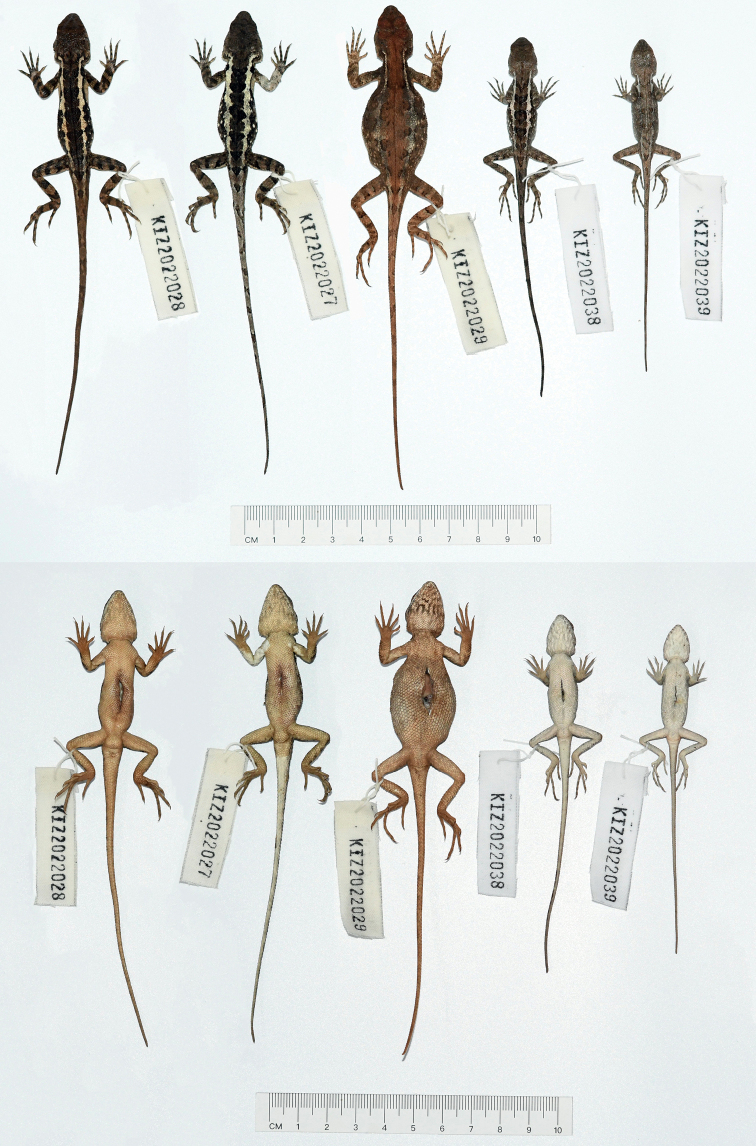
Dorsal view (upper) and ventral view (lower) of the type series of *Diplodermatachengense* sp. nov. in preservative.

**Figure 14.**
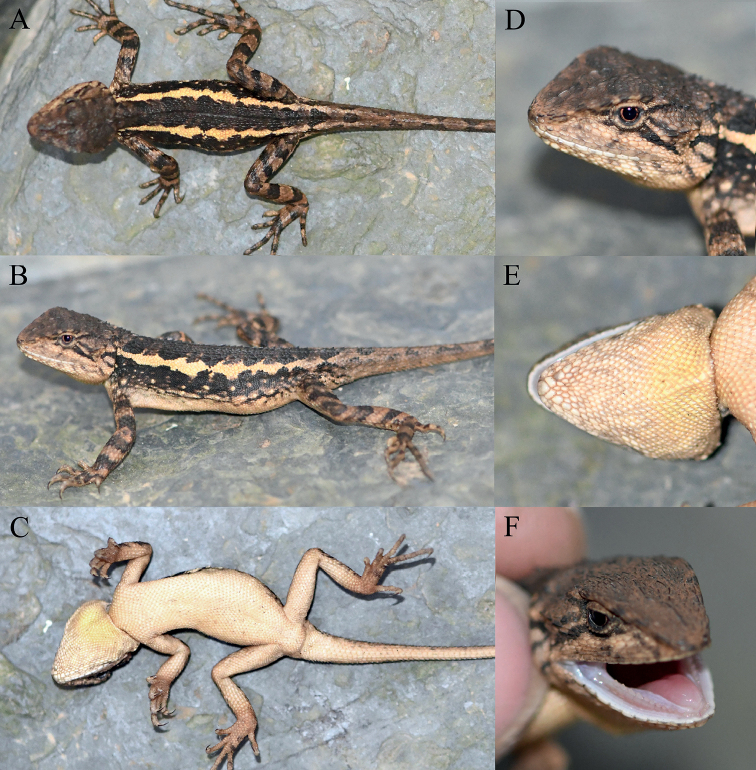
Holotype (KIZ2022028) of *Diplodermatachengense* sp. nov. in life **A** dorsal view **B** lateral view **C** ventral view **D** close-up view of the dorsolateral side of the head **E** close-up view of the ventral side of the head **F** close-up view of the oral cavity.

**Figure 15.**
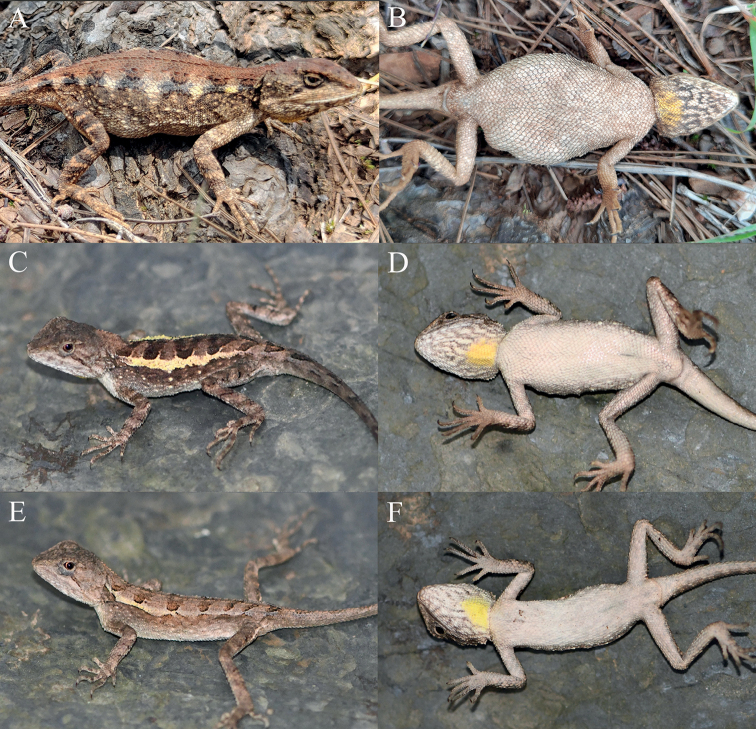
Paratypes of *Diplodermatachengense* sp. nov. in life **A, B** the female paratype KIZ2022029 **C, D** the subadult male paratype KIZ2022038 **E, F** the subadult female paratype KIZ2022039.

#### Variations.

The variations of metrical characteristics of the type series are provided in Table 5. Other variations are as follows: the dorsal colour is paler, there are some dark inverted triangular patterns between the two dorsolateral stripes, the radial stripes around eyes are more indistinct, and the dark stripes on ventral head are more distinct in all paratypes; in addition, the dorsolateral stripes are indistinct, yellowish white in the female paratypes.

**Table 5.** Morphological data of the type series of *Diplodermatachengense* sp. nov. Morphometric measurements are in the unit of mm. For measurement and count methods and abbreviations, see the Materials and methods.

	KIZ2022028 holotype ♂	KIZ2022027 paratype ♂	KIZ2022029 paratype ♀	KIZ2022038 paratype subadult ♂	KIZ2022039 paratype subadult ♀
SVL	55.2	55.7	65.4	42.7	40.6
TAL	104.5	104.8	102.1	81.6	73.6
HL	17.5	17.7	18.4	13.9	12.6
HW	12.7	14.1	13.6	10.3	9.2
HD	11.1	10.9	11.8	8.8	7.7
SEL	6.3	6.2	6.4	5.1	4.5
FLL	25.9	25.0	30.2	21.8	19.6
HLL	41.7	43.6	45.6	36.4	33.1
T4L	10.2	9.4	10.9	9.0	8.5
TRL	24.1	25.1	33.1	18.7	17.9
TAL/SVL	1.89	1.88	1.56	1.91	1.81
SEL/HL	0.36	0.35	0.35	0.37	0.36
HW/HL	0.73	0.80	0.74	0.74	0.73
HD/HW	0.87	0.77	0.87	0.85	0.84
FLL/SVL	0.47	0.45	0.46	0.51	0.48
HLL/SVL	0.76	0.78	0.70	0.85	0.82
TRL/SVL	0.44	0.45	0.51	0.44	0.44
SL	9/9	8/8	9/9	9/9	8/9
IL	10/10	9/9	11/10	9/10	10/9
NSL	1/1	1/1	1/1	1/1	1/1
MD	43	38	39	44	38
F4S	16/17	16/15	19/19	16/16	17/16
T4S	21/22	21/21	24/23	20/20	21/20
SOR	4/4	4/4	4/4	4/4	4/4
VN	55	51	56	51	53

#### Comparisons.

*Diplodermatachengense* sp. nov. differs from *D.brevipes*, *D.chapaense*, *D.fasciatum*, *D.hamptoni*, *D.luei*, *D.makii*, *D.menghaiense*, *D.micangshanense*, *D.ngoclinense*, *D.polygonatum*, *D.swinhonis*, and *D.yunnanense* by the presence of a transverse gular fold (vs. absence).

*Diplodermatachengense* sp. nov. differs from *D.dymondi*, *D.panlong*, *D.slowinskii*, *D.varcoae*, and *D.swild* by having concealed tympana (vs. exposed).

*Diplodermatachengense* sp. nov. differs from *D.drukdaypo*, *D.flaviceps*, *D.shuoquense*, *D.splendidum*, and *D.vela* by the presence of a distinct gular spot in males in life (vs. absence).

*Diplodermatachengense* sp. nov. differs from *D.batangense*, *D.bowoense*, *D.brevicauda*, *D.daochengense*, *D.flavilabre*, *D.formosgulae*, *D.iadinum*, *D.laeviventre*, *D.limingensis*, *D.xinlongense*, *D.yangi*, *D.yongshengense*, *D.yulongense*, and *D.zhaoermii* by having a pale yellow gular spot in males in life (vs. chartreuse, blue, green, lilac, orange, or yellowish white).

*Diplodermatachengense* sp. nov. differs from *D.angustelinea* by wide strongly jagged dorsolateral stripes in males (vs. narrow, feebly jagged); *D.grahami* by having relatively longer hind limbs (HLL/SVL 0.70–0.78 vs. 0.61), having a distinct transverse gular fold (vs. feeble), and the presence of dorsolateral stripes (vs. absence); from *D.kangdingense* by the absence of skin folds under nuchal and dorsal crests in males (vs. presence), and having white ventrolateral surface of body in males in life (vs. yellow); from *D.panchi* by having relatively longer hind limbs in females (HLL/SVL 0.70 vs. 0.60–0.66), the presence of a distinct gular spot in females in life (vs. absence), and the presence of black stripes on ventral head (vs. absence); and from *D.qilin* by having a relatively shorter tail (TAL/SVL 1.88–1.89 vs. 2.01–2.18 in males, 1.56 vs. 1.74–2.00 in females) and having a pink tongue (vs. pale flesh coloured).

*Diplodermatachengense* sp. nov. is phylogenetically sister to *D.aorun*; however, *Diplodermatachengense* sp. nov. can be differentiated from the latter by having a pale yellow gular spot in both sexes in life (vs. blue in both sexes), having a relatively much shorter tail (TAL/SVL 1.88–1.89 vs. 2.12–2.21 in males, 1.56 vs. 1.91–2.08 in females), having a greater ratio of head width to head length (HW/HL 0.73–0.80 vs. 0.68–0.73 in males, 0.74 vs. 0.67–0.70 in females), and the absence of skin folds under nuchal and dorsal crests in males (vs. presence).

*Diplodermatachengense* sp. nov. differs from *Diplodermadanbaense* sp. nov. by the presence of a distinct gular spot in both sexes in life (vs. absence), the presence of distinct radial stripes around eyes (vs. absence), the absence of distinct, dark, hollow, approximately rhomboid patterns between dorsolateral stripes on dorsum (vs. absence), and the absence of skin folds under nuchal and dorsal crests in males (vs. presence).

*Diplodermatachengense* sp. nov. differs from *Diplodermadonglangense* sp. nov. by having vermiculate stripes on ventral head (vs. short black stripes), having strongly jagged dorsolateral stripes in males (vs. moderately jagged), and the presence of distinct radial stripes around eyes (vs. absence).

*Diplodermatachengense* sp. nov. differs from *Diplodermajiulongense* sp. nov. by having a relatively shorter tail (TAL/SVL 1.56–1.89 vs. 2.33–2.71), the presence of vermiculate stripes on ventral head (vs. absence), and having wide, strongly jagged dorsolateral stripes in males (vs. narrow, smooth edged).

#### Distribution.

This species is currently known only from its type locality in Tacheng Town, Weixi County, Diqing Prefecture, Yunnan Province, China (Fig. 1).

#### Natural history.

**Figure 16.**
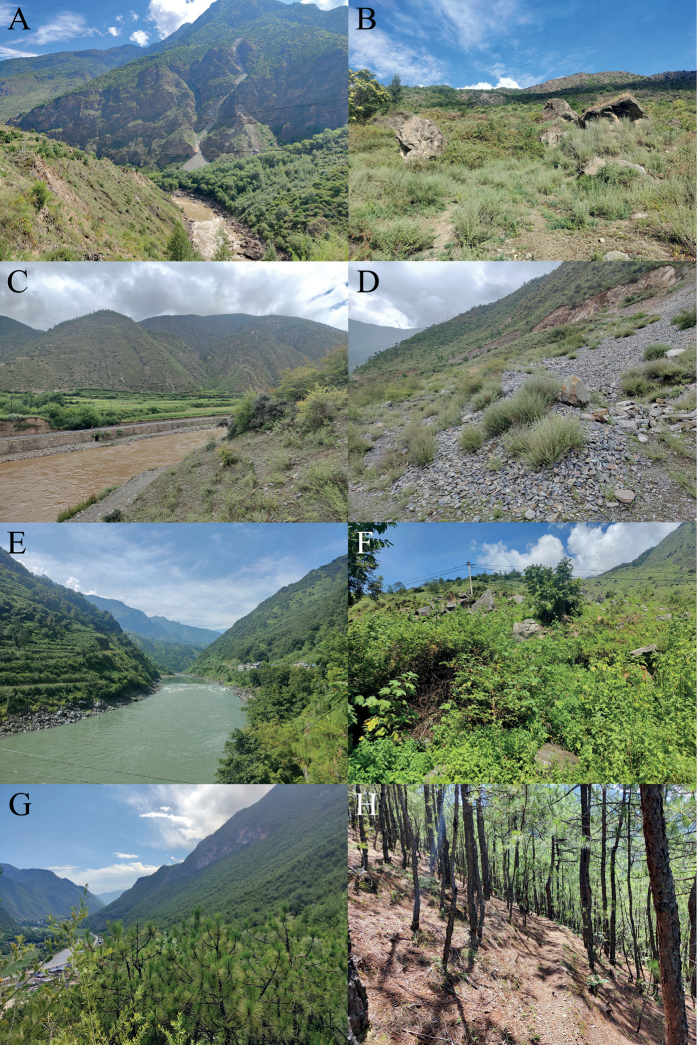
Habitats of the new species **A** distant view of the type locality of *Diplodermadanbaense* sp. nov. **B** close view of the type locality of *Diplodermadanbaense* sp. nov. **C** distant view of the type locality of *Diplodermadonglangense* sp. nov. **D** close view of the type locality of *Diplodermadonglangense* sp. nov. **E** distant view of the type locality of *Diplodermajiulongense* sp. nov. **F** close view of the type locality of *Diplodermajiulongense* sp. nov **G** distant view of the type locality of *Diplodermatachengense* sp. nov. **H** close view of the type locality of *Diplodermatachengense* sp. nov.

All specimens were collected between 1 and 5 p.m. on the ground in the secondary coniferous forest (Fig. 16G, H) beside the Lapu River, which is a tributary of the Jinsha River.

## ﻿Discussion

With a rapidly increasing number of species, the genus *Diploderma* consists of 42 species in total, whereof nearly half were described in the last five years, and more than one third were described in the last three years (e.g., Cai et al. 2022; Liu et al. 2022; Uetz et al. 2022). Together with the four new species described in this study, we bring the total number of species in this genus to 46.

Among the four new species described in this study, *Diplodermajiulongense* sp. nov. and *Diplodermatachengense* sp. nov. have relatively small genetic distances (2.9% and 2.8%) from the closely related species *D.angustelinea* and *D.aorun*. However, there are significant morphological differences between *Diplodermajiulongense* sp. nov. and *D.angustelinea* and between *Diplodermatachengense* sp. nov. and *D.aorun*. *Diplodermajiulongense* sp. nov. differs from *D.angustelinea* by having a relatively much longer tail, relatively longer fore-limbs, relatively longer hind limbs, and having smooth edged dorsolateral stripes in males. *Diplodermatachengense* sp. nov. differs from *D.aorun* by having an obviously differently coloured gular spot in life, having a relatively shorter tail, and the absence of skin folds under nuchal and dorsal crests in males in life. In addition, the genetic distances between them and their closely related species are both greater than that (2.6%) between the two recognized species *D.drukdaypo* and *D.vela*. Although the genetic distance is small between *D.drukdaypo* and *D.vela*, they have distinct and stable morphological differences (i.e., relative limb length, relative tail length, and keeling of ventral body scales; Wang et al. 2015, 2019b). Thus, they are regarded as two distinct species each that occupy distinct sections of the Mekong River and live at different elevations (Wang et al. 2021b). Therefore, taking the case of the species pair of *D.drukdaypo* and *D.vela* as the calibration, *Diplodermajiulongense* sp. nov. and *Diplodermatachengense* sp. nov. also have surpassed intraspecific variation level and reached a specific status.

Another new species described in this study, *Diplodermadanbaense* sp. nov., is at first glance similar in morphology to *D.flaviceps*. To compare morphological characteristics between *Diplodermadanbaense* sp. nov. and *D.flaviceps*, we examined 27 topotypic or near-topotypic specimens of *D.flaviceps* (Suppl. material 2), and found several non-overlapping morphological characteristics (i.e., relative tail length, relative hind limb length, and relative head width) between both taxa (see Table 6). In addition, the genetic distance (4.4%) between *Diplodermadanbaense* sp. nov. and *D.flaviceps* is approximate to that (4.1%) between *D.brevicauda* and *D.qilin*, greater than that (3.2%) between *D.formosgulae* and *D.drukdaypo* and that (3.3%) between *D.formosgulae* and *D.vela*, and much greater than that (2.6%) between *D.drukdaypo* and *D.vela*. *Diplodermabrevicauda* and *D.qilin* are similar in morphology, according to Wang et al. (2021b), there are only differences in relative tail length, relative hind limb length and oral cavity colour between them, and these differences are considered sufficient to distinguish different species (Wang et al. 2021b).

**Table 6.** Comparison between *Diplodermadanbaense* sp. nov. and *D.flaviceps*. For morphological abbreviations see the Materials and methods. Detailed measurements and pholidosis data for each examined specimen of *D.flaviceps* are given in Suppl. material 2.

	*Diplodermadanbaense* sp. nov.		*Diplodermaflaviceps*	
	♂ (*n* = 4)	♀ (*n* = 1)	♂ (*n* = 18)	♀ (*n* = 9)
SVL	68.7–77.0	76.6	60.5–75.6	53.4–67.1
TAL	112.7–130.0	119.1	117.7–151.0	103.7–127.0
HL	21.4–26.6	23.5	18.9–24.9	16.3–20.5
HW	15.0–17.9	14.7	14.6–20.6	12.6–15.6
HD	12.8–15.0	12.8	10.7–14.8	9.6–12.5
SEL	7.5–9.9	8.4	5.9–8.9	5.2–6.8
FLL	28.7–33.1	33.6	28.8–36.1	24.3–31.6
HLL	49.1–55.2	52.1	48.1–60.0	39.2–50.9
T4L	12.2–12.8	12.5	11.4–14.9	9.6–11.7
TRL	29.2–33.2	34.5	26.7–34.6	24.3–33.0
TAL/SVL	1.61–1.78	1.55	1.88–2.09	1.73–2.17
HL/SVL	0.31–0.35	0.31	0.31–0.34	0.29–0.33
HW/HL	0.66–0.75	0.63	0.76–0.84	0.71–0.78
HD/HW	0.80–0.85	0.87	0.70–0.78	0.75–0.83
FLL/SVL	0.41–0.47	0.44	0.44–0.49	0.43–0.49
HLL/SVL	0.66–0.70	0.65	0.72–0.80	0.70–0.81
T4L/SVL	0.16–0.18	0.16	0.18–0.21	0.17–0.21
SL	9–10	10–11	8–11	9–11
IL	10–11	11	10–13	10–12
NSL	1–2	2	1–2	1–2
MD	47–53	58	40–55	46–54
F4S	16–20	19–20	16–21	16–19
T4S	21–25	24–26	22–26	22–27
SOR	4–5	4–5	3–5	3–5

Allopatric distribution across the Hengduan Mountain Region have been suggested to create reproductive isolation through geographic isolation (Wang et al. 2015, 2021a; Dong et al. 2020). Wang et al. (2021b) considered that *D.drukdaypo* may represent a recently diverged species from the *D.vela* lineage, adapted to higher-elevated habitats in the upper Mekong River valley, and suggested that further studies are needed to gain a better understanding of gene flow and speciation mechanisms between these two sister species. Although *D.formosgulae* is morphologically similar and genetically close to *D.drukdaypo* and *D.vela*, Wang et al. (2021a) stated that *D.formosgulae* possesses an allopatric distribution with the latter two species and represents a recently diverged lineage, evolutionarily distinct. Likewise, *Diplodermadanbaense* sp. nov. also possesses an allopatric distribution with *D.flaviceps* and may represent a recently diverged species, adapted to higher-elevated habitats in the upper Dadu River valley.

To further confirm the validity of these three new species, we conducted detailed surveys in the field to verify whether there is geographical isolation between these three new species and their closely related relatives. At first, the type locality of *Diplodermatachengense* sp. nov. (at 2180 m elevation) is approximately 100 km downstream along the Jinsha River from the type locality of *D.aorun* (at 2198 m elevation). Although the altitudinal ranges are similar, their habitats are completely different, one is a hot-dry valley of the Jinsha River, the other is a distant forested region. Therefore, there is a clear geographical and ecological isolation between the populations of *Diplodermatachengense* sp. nov. and *D.aorun*. Further, the type locality of *Diplodermajiulongense* sp. nov. (at 1680 m elevation) is approximately 130 km downstream along the Yalong River from the type locality of *D.angustelinea* (at 2017 m elevation). Although the linear distance between the two localities is short, there is a branch of the Daxue Mountain, with the highest peak at approximately 5000 m elevation, between them, serving as a clear geographic barrier. In addition, there is another population of *Diploderma* (D.cf.daochengense) occupying part valley section of the Yalong River, which bypasses the branch of the Daxue Mountain, between the type localities of *Diplodermajiulongense* sp. nov. and *D.angustelinea*. At last, the type locality of *Diplodermadanbaense* sp. nov. (at 2020 m elevation) is approximately 150 km upstream along the Dadu River from the type locality of *D.flaviceps* (at 1300 m elevation). Although there is no obvious geographical barrier between the two localities except for the altitudinal difference, we have not found any transitional type individuals between *Diplodermadanbaense* sp. nov. and *D.flaviceps*, nor have we found *Diplodermadanbaense* sp. nov. and *D.flaviceps* occurring in sympatry. In addition, the relatively great genetic distance (4.4%) between *Diplodermadanbaense* sp. nov. and *D.flaviceps* also supports an isolation process. A similar situation occurs between the two recognized closely related species *D.panlong* and *D.swild*. Through our observation, we found that these two species are similar in morphology and colouration, their habitats and natural history are also similar, as both inhabit forests or forest margins and both are more arboreal than terrestrial, and there is no obvious geographical barrier between them. However, these two species have not been found in sympatry, and there is a great genetic distance between them. We speculate that some historical reasons may have led to the isolation of different populations, thus, they lost genetic exchange and differentiated into different species.

Finally, the mitochondrial ND2 gene is considered to be able to better distinguish different species and has been widely used in phylogenetic analyses of Agamidae (i.e., Zug et al. 2006; Grismer et al. 2016; Ambekar et al. 2020; Liu et al. 2021; Wang et al. 2022a), and the phenomenon of different species with small genetic distances in the ND2 gene is well known from other genera of Agamidae, such as in the genus *Sitana* Cuvier, where a genetic distance of 3–4% in the ND2 gene is considered sufficient to distinguish different species (Ambekar et al. 2020). This supports our taxonomic actions presented herein, viz. that different populations with relatively small genetic distances in the ND2 gene can represent different species in the Agamidae.

## Supplementary Material

XML Treatment for Diploderma danbaense

XML Treatment for Diploderma donglangense

XML Treatment for Diploderma jiulongense

XML Treatment for Diploderma tachengense

## Supplementary materials

Supplementary material 1 Uncorrected genetic pairwise distances (*p*-distances) (%) between species based on the mitochondrial ND2 gene sequences. Data type table (excel document) https://binary.pensoft.net/file/812098This dataset is made available under the Open Database License (http://opendatacommons.org/licenses/odbl/1.0/). The Open Database License (ODbL) is a license agreement intended to allow users to freely share, modify, and use this Dataset while maintaining this same freedom for others, provided that the original source and author(s) are credited.Shuo Liu, Mian Hou, Natalia B. Ananjeva, Dingqi Rao

Supplementary material 2 Detailed morphological measurements and pholidosis characteristics for each examined specimen of *Diplodermaflaviceps*. All measurements are in the unit of mm. “–” indicates not available. For measurement and count methods and abbreviations, see the Materials and methods. Data type table (excel document) https://binary.pensoft.net/file/812099This dataset is made available under the Open Database License (http://opendatacommons.org/licenses/odbl/1.0/). The Open Database License (ODbL) is a license agreement intended to allow users to freely share, modify, and use this Dataset while maintaining this same freedom for others, provided that the original source and author(s) are credited.Shuo Liu, Mian Hou, Natalia B. Ananjeva, Dingqi Rao
